# Supplementary figures and images for: Large-scale docking predicts that sORF-encoded peptides may function through protein-peptide interactions in Arabidopsis thaliana
Source: PLoS One. 2018 Oct 15;13(10):e0205179. doi: 10.1371/journal.pone.0205179 (PMC6188750; doi:10.1371/journal.pone.0205179)

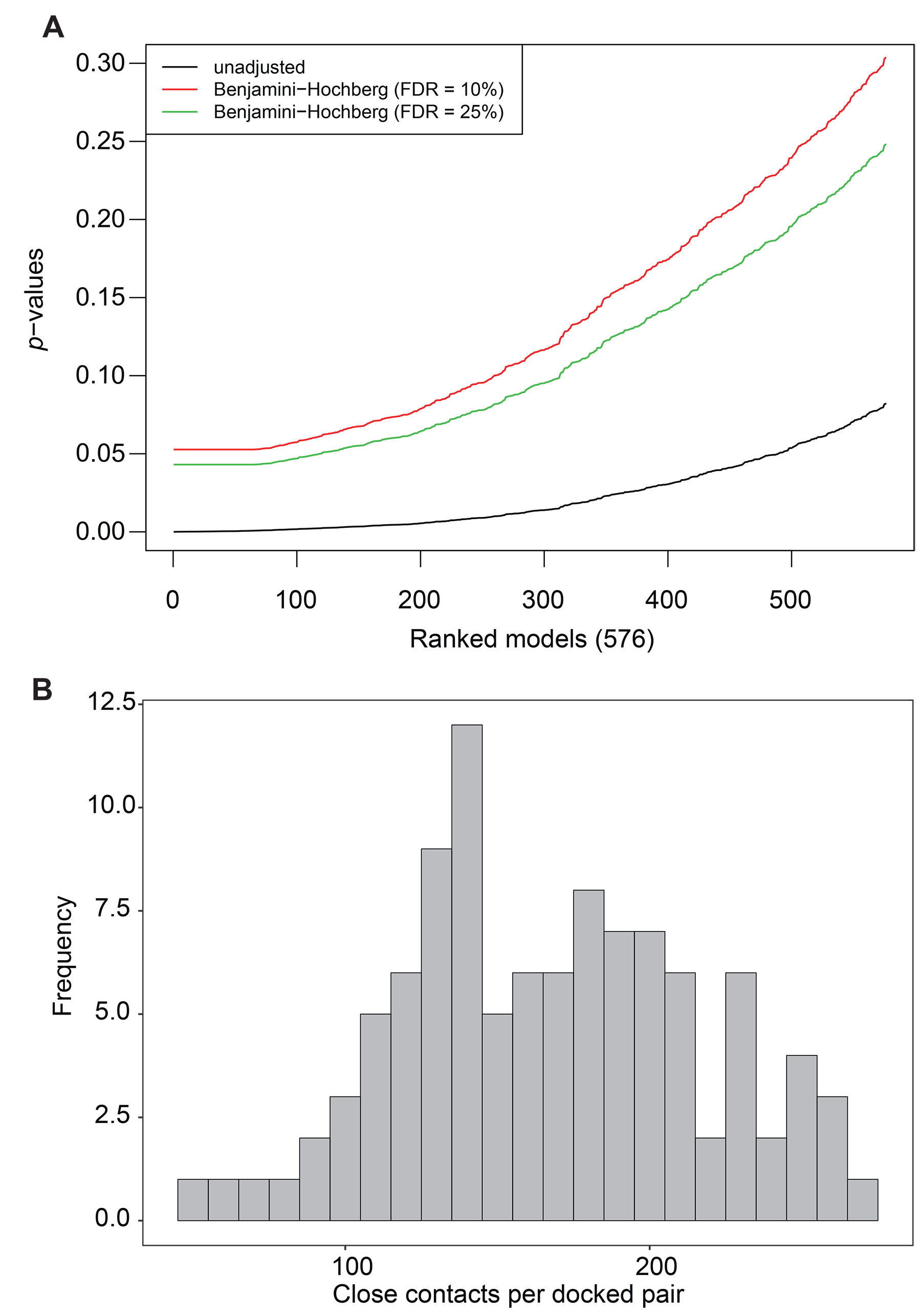

Supplement: S1 Fig — (A) Plot of adjusted p-values using Benjamini-Hochberg correction method vs. raw p-values for the 576 docked models. (B) Histogram showing distribution of the number of close contacts across the 104 top models. (TIF) [file pone.0205179.s001.tif]

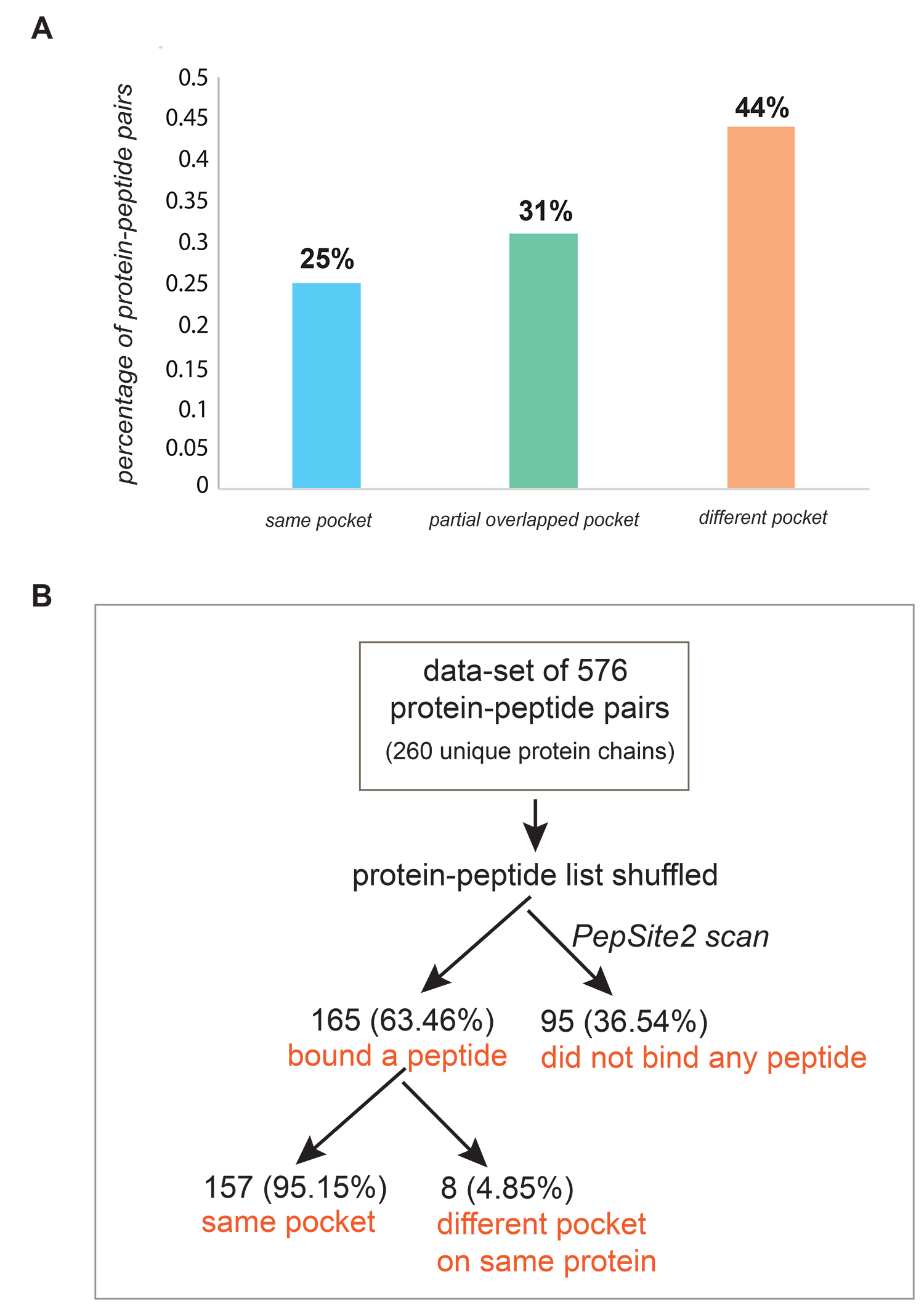

Supplement: S2 Fig — (A) Comparison of pepATTRACT-local and blind docking protocols (B) Effect of random shuffling on the binding of peptides to pockets (TIF) [file pone.0205179.s002.tif]

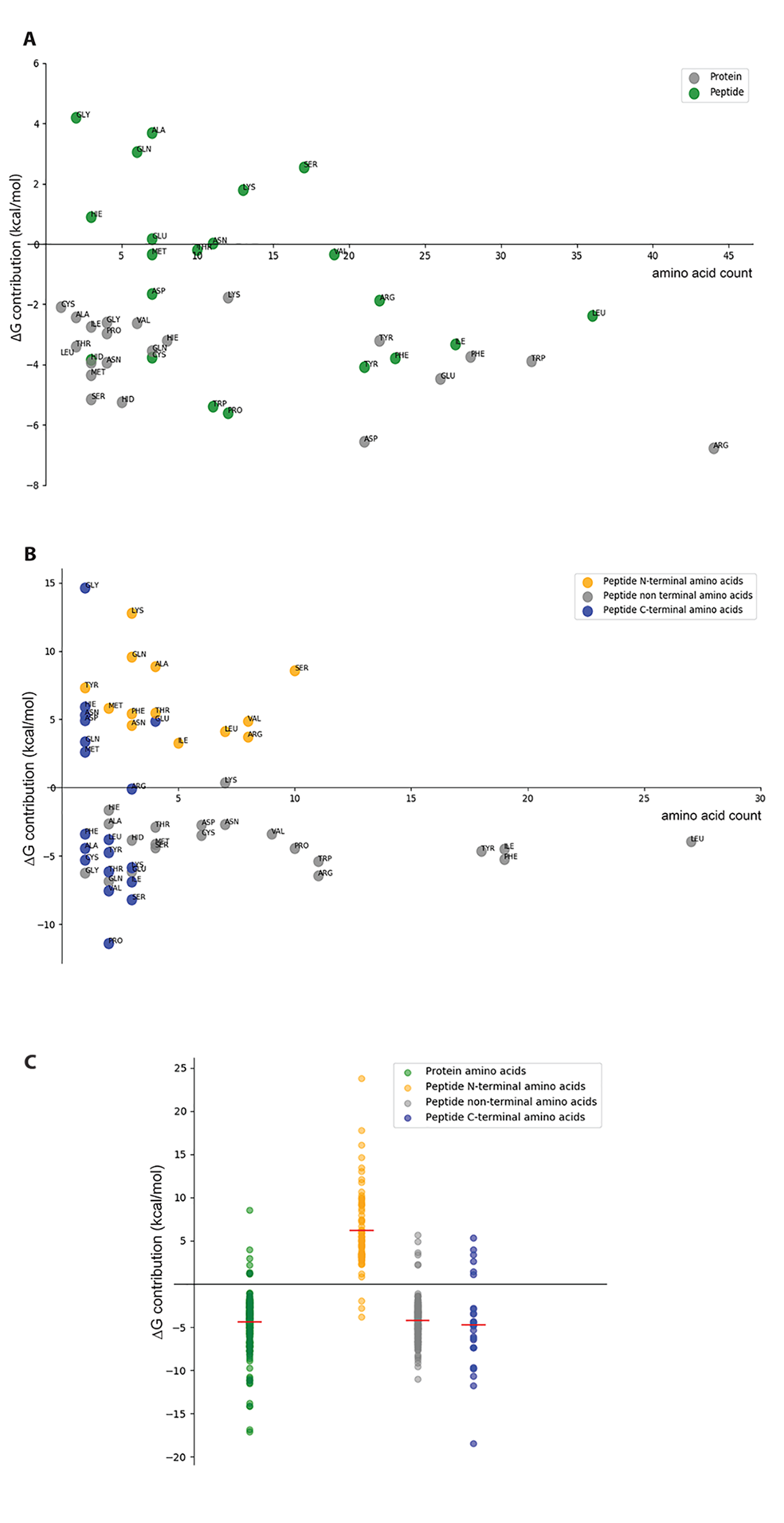

Supplement: S3 Fig — (A) Average contributions to the binding energy for each amino acid type, for peptide and protein amino acids separately, and (B) for peptide amino acids at different locations within the peptides. (C) Individual data points for all amino acids, from which the averages were made, with red lines representing the average values. The represented data includes only amino acids whose binding contribution is at least 40% of the maximal contribution value within the respective system. (TIF) [file pone.0205179.s003.tif]

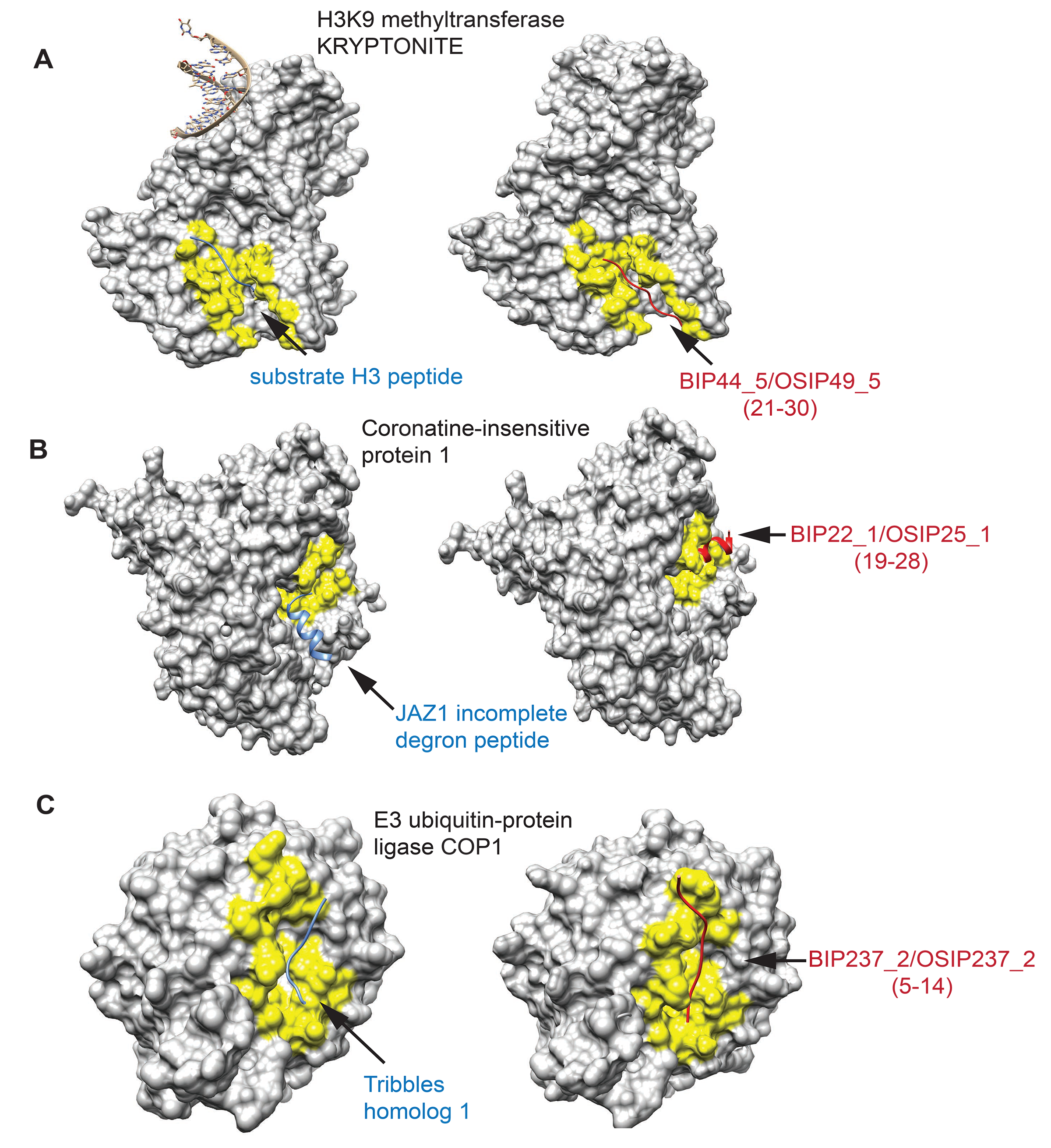

Supplement: S4 Fig — The peptide binding pocket is highlighted in yellow. (TIF) [file pone.0205179.s004.tif]
