# Supplementary material for: Large-scale docking predicts that sORF-encoded peptides may function through protein-peptide interactions in Arabidopsis thaliana
Source: PLoS One. 2018 Oct 15;13(10):e0205179. doi: 10.1371/journal.pone.0205179 (PMC6188750; doi:10.1371/journal.pone.0205179)
Supplement: S1 Table — (PDF) [file pone.0205179.s005.pdf]

Ligand	SIP	PDB_match	PDB Chain	Raw *p*-values (PepSite2)	BH correction (FDR=0.25)
QGQVT	BIP38_4/OSIP43_4	5ECP	F	0.0000067	0.043062533
FNRP	BIP154_5/OSIP145_5	2O01	A	0.0000355	0.043062533
SFAQF	BIP171_6/OSIP158_6	5KSD	B	0.0000550	0.043062533
KNPHQFL	BIP232_2/OSIP232_2	5KSD	B	0.0000595	0.043062533
ELLQQQRF	BIP243_5/OSIP243_5	5GIJ	B	0.0000674	0.043062533
WLVTGQT	OSIP166_1	5ECK	A	0.0000794	0.043062533
WLGP	OSIP168_1	4PUT	A	0.0000907	0.043062533
LGRIPG	BIP47_6	3FY4	B	0.0001067	0.043062533
HPFLVCWVSS	BIP47_6	3FY4	A	0.0001085	0.043062533
NNGPNPTVSV	OSIP164_1	4KA7	A	0.0001091	0.043062533
SAQPQELLPQ	BIP242_4/OSIP242_4	2VTB	F	0.0001209	0.043062533
SISKACERIP	OSIP4_4	2Q4X	B	0.0001221	0.043062533
TLSLVAFYFA	BIP232_1/OSIP232_1	2WSE	B	0.0001258	0.043062533
HCSPNLNIVH	BIP244_8/OSIP244_8	3FY4	B	0.0001346	0.043062533
YPVLDAVEGA	BIP47_6	3FY4	C	0.0001355	0.043062533
QNQIKEYFIS	BIP244_8/OSIP244_8	3FY4	A	0.0001362	0.043062533
MGPFSGPSEV	OSIP168_1	4KA7	A	0.0001385	0.043062533
WSMFHGYNST	BIP244_4/OSIP244_4	2WSC	B	0.0001406	0.043062533
WIHICMRRET	BIP38_4/OSIP43_4	5ECM	B	0.0001497	0.043062533
IGFGMKASPT	BIP52_1	3FY4	C	0.0001561	0.043062533
DSTNICARGS	OSIP4_4	2Q4X	B	0.0001653	0.043062533
IHHPFRRCLF	BIP216_1/OSIP216_1	3FY4	A	0.0001842	0.043062533
RSAVATWRQL	BIP232_1/OSIP232_1	2O01	B	0.0001937	0.043062533
SSPARPQHLL	BIP223_6/OSIP223_6	4LIX	A	0.0002093	0.043062533
APWILTVAAS	OSIP164_1	4KA7	A	0.0002172	0.043062533
YTFICCTISI	BIP244_4/OSIP244_4	2WSE	B	0.0002213	0.043062533
VYRQMAQFTL	BIP90_3	4E2S	L	0.0002269	0.043062533
SILFFPLCKF	BIP171_6/OSIP158_6	5KSD	B	0.0002515	0.043062533
ALRIPKLYLV	OSIP85_3	2WSE	B	0.0002528	0.043062533
YKQSFACLAS	BIP142_3/OSIP134_3	2VTB	E	0.0002532	0.043062533
NNKAKISIVC	BIP216_1/OSIP216_1	3FY4	C	0.0002677	0.043062533
SLQP	BIP213_1/OSIP213_1	5LAL	B	0.0002713	0.043062533
SKNHR	BIP244_4/OSIP244_4	2WSF	B	0.0002800	0.043062533
VMTNGNSSNR	OSIP4_4	2Q4X	B	0.0002956	0.043062533
DISHMPYRFK	BIP243_5/OSIP243_5	5GR9	B	0.0002976	0.043062533
PKTHLI	BIP149_5/OSIP141_5	4LIX	A	0.0003328	0.043062533
SSDPVNIWKE	BIP235_4/OSIP235_4	5GQR	B	0.0003576	0.043062533
EQYWLPLLVD	OSIP51_2	5ECN	A	0.0003631	0.043062533
HHRTRAGRTF	OSIP51_2	5ECK	A	0.0003680	0.043062533
INKRMWLIKL	BIP38_4/OSIP43_4	5ECP	F	0.0003692	0.043062533
CSNSHQW	BIP230_5/OSIP230_5	5ECK	A	0.0003772	0.043062533
FLYFIFFSFS	BIP140_6/OSIP130_6	2VTB	E	0.0003950	0.043062533
KAYDRVRKKV	BIP52_1	3FY4	C	0.0004003	0.043062533
GSFFKLVESR	BIP139_6	4PUT	A	0.0004064	0.043062533
DLASDNRNPL	BIP73_5/OSIP75_5	2VTB	E	0.0004161	0.043062533
PFAQ	BIP22_1/OSIP25_1	3OGK	L	0.0004249	0.043062533

Ligand	SIP	PDB_match	PDB Chain	Raw *p*-values (PepSite2)	BH correction (FDR=0.25)
KAPD	BIP180_2/OSIP167_2	3T33	A	0.0004302	0.043062533
TFLG	BIP224_3/OSIP224_3	2Q3O	B	0.0004323	0.043062533
TLTTEPTMVI	BIP243_5/OSIP243_5	5GQR	B	0.0004362	0.043062533
WITYLRRVMD	BIP244_8/OSIP244_8	3FY4	C	0.0004377	0.043062533
APCPSLLHKL	BIP209_3/OSIP209_3	5IYX	A	0.0004596	0.043062533
FNHS	BIP93_2/OSIP89_2	4KA8	A	0.0004653	0.043062533
NFHLPQP	BIP171_2/OSIP158_2	2Q4E	B	0.0004972	0.043062533
HTYKLFCKNI	BIP40_1/OSIP44_1	2VTB	E	0.0005154	0.043062533
VQYILSTHQH	BIP230_5/OSIP230_5	5ECK	A	0.0005673	0.043062533
YFSMQVFYEF	BIP243_5/OSIP243_5	5GIJ	B	0.0005711	0.043062533
KGILTVQSAG	OSIP164_1	4KA8	A	0.0005740	0.043062533
QYRVSDFALP	BIP89_6	5I32	A	0.0006018	0.043062533
LDRISKAQSV	BIP139_6	4PUT	A	0.0006089	0.043062533
RYCMRLMMLN	BIP86_6	3PYA	A	0.0006120	0.043062533
VPCSINYGLV	BIP242_4/OSIP242_4	2VTB	A	0.0006601	0.043062533
VRGNNVTTHV	OSIP168_1	4KA7	A	0.0006669	0.043062533
FSYQLSDFVC	BIP139_6	4PUT	A	0.0007097	0.043062533
TTQALHLTIL	OSIP166_1	5ECK	A	0.0007109	0.043062533
ILSHLQALPH	BIP90_3	4E2S	L	0.0007477	0.043072353
EQVHSL	BIP209_3/OSIP209_3	5IYX	A	0.0007776	0.043103984
LFLRFYNLNS	BIP154_5/OSIP145_5	2O01	A	0.0008134	0.043167944
ISCGHKSHIF	BIP230_5/OSIP230_5	5ECK	A	0.0008148	0.043167944
PNGREYIFYR	BIP231_3/OSIP231_3	3NME	B	0.0008441	0.04324374
GRECLSQRIM	BIP208_2/OSIP208_2	2XQR	K	0.0008556	0.04328148
AFNSHFLNQS	BIP231_4/OSIP231_4	4IH4	A	0.0008715	0.043322345
IAIGAFHAMA	OSIP164_1	4KA8	A	0.0009465	0.043548975
DKVQRRGTVC	BIP59_6	2XQR	A	0.0009545	0.043576299
LNKICIFFST	BIP154_5/OSIP145_5	2O01	A	0.0009756	0.043653769
SFYIDLGPHS	BIP93_2/OSIP89_2	4KA7	A	0.0009872	0.043705064
KKPFVAFECP	OSIP51_4	2FGE	A	0.0010040	0.043756402
KHFQFHYKIS	BIP213_1/OSIP213_1	5LAL	B	0.0010060	0.043763962
QIKPQRT	BIP89_4	5GZZ	C	0.0010220	0.043822401
SSSRSNQNNT	BIP240_6/OSIP240_6	5ECK	A	0.0010260	0.04384527
HTQRQQKSNI	BIP222_1/OSIP222_1	5KOR	A	0.0011030	0.044133164
VQVGKYLPPE	BIP31_5	2FGE	A	0.0011450	0.044315625
LCSEIQPTTS	BIP224_3/OSIP224_3	2Q3O	A	0.0011880	0.044487676
NINKSKYSLS	OSIP4_4	2Q4X	B	0.0012390	0.044724416
LLFLSFYFSC	BIP140_6/OSIP130_6	2VTB	B	0.0012600	0.044804434
VPPSQPSPCH	BIP38_2/OSIP43_2	3CQN	B	0.0013030	0.044975634
KKAYGKIDMH	BIP93_2/OSIP89_2	4KA7	A	0.0013570	0.045209824
RFFQFARKQL	BIP240_7/OSIP240_7	1U1U	A	0.0013590	0.045209824
NFHVTKDTSC	BIP116_3	2O01	A	0.0013640	0.045245995
FYIPSNFVYY	BIP229_2/OSIP229_2	2Q3O	B	0.0013750	0.045275017
GSRFHECSS	BIP232_1/OSIP232_1	2WSE	B	0.0014120	0.045504712
VSLVTIPNNN	OSIP108_3	5HAD	A	0.0014660	0.0457405
TQSVPFTCKS	BIP231_2/OSIP231_2	2P1N	B	0.0014710	0.045756756

Ligand	SIP	PDB_match	PDB Chain	Raw *p*-values (PepSite2)	BH correction (FDR=0.25)
RRKSTNQMGL	BIP210_2/OSIP210_2	4TNM	A	0.0015520	0.046163729
PLSTFSLFFF	BIP180_1/OSIP167_1	5HAD	A	0.0015630	0.046219798
MTHTFSPSAC	OSIP108_3	5HAD	A	0.0016030	0.046427251
INGWIHELEL	BIP140_6/OSIP130_6	2VTB	B	0.0016380	0.046580018
TFEQYWLPLL	OSIP51_2	4A0H	A	0.0016520	0.046648252
LAEDTFGEIS	BIP142_3/OSIP134_3	2VTB	D	0.0016740	0.046760405
YSHEAAFPSA	BIP231_1/OSIP231_1	4TNM	A	0.0016970	0.046881324
RPGLRPNGPS	OSIP77_2	1WE9	A	0.0016970	0.046881324
SIGYLHFKNR	BIP149_5/OSIP141_5	4LIX	A	0.0017520	0.047145635
GMLFNRVKSH	BIP86_1	4LIX	A	0.0018840	0.047780464
RRCLFCFFPL	BIP216_1/OSIP216_1	1U3C	A	0.0018900	0.047812348
IVTLFLI	BIP231_2/OSIP231_2	2P1N	B	0.0018980	0.047862954
PLSQKKENHF	BIP232_2/OSIP232_2	5KSD	A	0.0019120	0.047922529
KAWSSRHTLL	BIP73_5/OSIP75_5	2VTB	A	0.0019290	0.048011395
AILDAAVIPA	BIP97_6	5KOR	C	0.0019610	0.048169944
PTIVPDCLSK	BIP235_4/OSIP235_4	5GIJ	B	0.0019780	0.048277539
NKKLIWPSWS	BIP180_2/OSIP167_2	3T33	A	0.0020080	0.048429206
NMLPWNATRI	BIP89_2	4EQ4	A	0.0020630	0.048706951
LRSMAACFAY	BIP89_6	5I32	A	0.0020630	0.048706951
VKLVDRRHVV	BIP139_6	4PUT	A	0.0021150	0.048965712
LLPSLAQRET	BIP3_1	4BQF	B	0.0021510	0.049151074
KRTVKGHMK	BIP155_6	4XK8	4	0.0021660	0.049229503
TKHCDQIYQD	BIP201_1/OSIP201_1	3T33	A	0.0022060	0.049426819
TTDKLIPEHM	BIP154_1/OSIP145_1	4DNU	A	0.0022100	0.049456892
PLVIAEMR	BIP208_2/OSIP208_2	2XQR	A	0.0022550	0.049686797
FVFLVLVVLL	BIP242_4/OSIP242_4	2VTB	B	0.0022930	0.049862601
PLDFHRVMMV	BIP208_5/OSIP208_5	1Q45	B	0.0023240	0.050037913
SHACTHNTY	OSIP68_6	3R0Q	G	0.0023360	0.050091036
LNVCNILLMY	BIP180_1/OSIP167_1	5HAD	A	0.0024900	0.050872606
ILRRICILIT	BIP231_6/OSIP231_6	4A0G	A	0.0025030	0.050945665
VGIVPRQKKV	OSIP70_3	1XQ1	A	0.0025320	0.051075564
NPSITLRRYV	BIP244_9/OSIP244_9	1XJ5	C	0.0025410	0.051123089
IDRWGRVLLQ	BIP88_2/OSIP84_2	2Q3O	A	0.0025620	0.051213665
KTSTRFCLIP	OSIP86_3	5DQR	A	0.0025930	0.051370006
WIIGLRLDKH	BIP116_3	2O01	A	0.0026140	0.051462632
FSFLNFQNWD	BIP228_1/OSIP228_1	2G0Q	A	0.0026190	0.051496988
VWINQILEQH	BIP214_1/OSIP214_1	1YHY	A	0.0026680	0.051725656
MWLIKLQGQV	BIP38_4/OSIP43_4	1XQ1	A	0.0026720	0.051737927
KDLTFFPPKN	BIP0_3	3RIZ	A	0.0027270	0.052016522
FPPSWIHHPF	BIP216_1/OSIP216_1	1U3C	A	0.0027270	0.052016522
NSSSSKPLSF	BIP224_3/OSIP224_3	2Q3O	A	0.0027680	0.052205995
TQTKLKCNTK	BIP222_1/OSIP222_1	5KOE	A	0.0027960	0.052317562
RFPIASAFSQ	BIP51_6	2I9Y	A	0.0029210	0.052894753
EREAYKEPCA	BIP231_1/OSIP231_1	4TNM	A	0.0029310	0.052951034
SNSHQW	BIP230_5/OSIP230_5	2Q42	A	0.0029320	0.052965514
DRSEIQFVVG	BIP59_6	2XQR	C	0.0030620	0.053585423

Ligand	SIP	PDB_match	PDB Chain	Raw *p*-values (PepSite2)	BH correction (FDR=0.25)
IFWWKRIRDN	BIP231_6/OSIP231_6	4A0G	A	0.0030770	0.053638742
GTPSEKEKLF	BIP208_5/OSIP208_5	2Q3O	A	0.0031080	0.053773093
TFQRNYECAS	BIP236_3/OSIP236_3	4N7Q	A	0.0031820	0.054111335
RRCVREVNDR	BIP142_3/OSIP134_3	2VTB	A	0.0032090	0.054222527
NKIYNFKILI	OSIP108_3	5HAD	A	0.0032360	0.054348325
GGDRGLYSGR	BIP140_6/OSIP130_6	2VTB	B	0.0032530	0.054441262
WLKSSVITYP	BIP209_2/OSIP209_2	3R0Q	G	0.0033190	0.054743787
SDLQKDSWVQ	BIP90_3	4E2Q	C	0.0033350	0.05480722
SQYYSPNHGY	OSIP52_3	2FGE	A	0.0033580	0.054907154
FLELPPYLDR	BIP31_5	2FGE	A	0.0033810	0.055019855
SFPNSNSGKV	BIP31_5	2FGE	B	0.0034090	0.055150462
SFPNSNSGKV	BIP31_5	2FGE	A	0.0034090	0.055150462
PKPQNHVFPR	BIP9_3	3OGK	D	0.0034090	0.055150462
RWCYPCRSSI	BIP223_3/OSIP223_3	3H7R	A	0.0034490	0.055332497
SKPAAGTCSR	OSIP86_3	5DQR	E	0.0034490	0.055332497
TISPFYEHNQ	BIP164_2	4Z63	A	0.0034550	0.055371644
QLPFLITDSN	BIP22_1/OSIP25_1	3OGK	F	0.0035550	0.055820995
TPLLSKQLHS	BIP245_4/OSIP245_4	5D79	A	0.0036090	0.056087942
FNTQSVPFTC	BIP231_2/OSIP231_2	3OGK	L	0.0037140	0.056594733
LGDHIARPRI	BIP88_2/OSIP84_2	1Q45	B	0.0037510	0.056772716
EKEIMNCSGF	BIP228_1/OSIP228_1	2G0Q	A	0.0038090	0.057055449
CFVCWVIGNE	BIP210_2/OSIP210_2	4TNM	A	0.0038990	0.057476199
GIYLYNKCAF	OSIP68_6	3R0Q	G	0.0039060	0.057508502
DHGLPFDTPA	BIP97_1	5E1J	A	0.0040120	0.057999618
QSGDGARQ	BIP26_4/OSIP30_4	1YBH	A	0.0040250	0.058056794
VNPSRF	OSIP99_1	1YDW	B	0.0040280	0.058088776
VHHESSNPSN	BIP229_2/OSIP229_2	2Q3O	A	0.0040530	0.058190034
FQQHKITRGH	BIP97_6	5KOR	A	0.0040530	0.058190034
LCSIEPERPL	OSIP77_6	2P1N	B	0.0040800	0.058302086
LLKQILTLVT	OSIP68_6	3R0Q	G	0.0041140	0.058441016
RCFPCHPS	BIP24_1	1RP0	A	0.0042420	0.059034087
FLMFSYFWCC	BIP6_6/OSIP8_6	1U3D	A	0.0042550	0.059077753
LSQRIMPLVI	BIP208_2/OSIP208_2	4L0Q	A	0.0043190	0.059352533
MIGEVKR	BIP88_2/OSIP84_2	2Q3O	A	0.0043280	0.059407385
FFDDVKSRRL	BIP103_3	2XQR	A	0.0043920	0.059654394
ISHLNRLPTR	BIP239_3/OSIP239_3	1U3D	A	0.0043920	0.059654394
FRGWDWVSEY	BIP31_2	2WSC	F	0.0044290	0.059827545
NHNTAPQSFS	BIP62_3/OSIP58_3	4RL5	B	0.0044440	0.059893749
KELQNTLTYI	BIP201_1/OSIP201_1	3T33	A	0.0044820	0.060052748
ALQNKLQRGP	BIP38_2/OSIP43_2	3CQR	A	0.0044970	0.060122348
RSHHRTRAGR	OSIP51_2	4A0G	A	0.0045120	0.060185628
GSESHVDATQ	BIP231_1/OSIP231_1	4TNM	A	0.0045200	0.060224296
SRFVLLPGMR	OSIP70_3	1XQ1	A	0.0045580	0.060391891
FAVEFQVRK	BIP212_2/OSIP212_2	5D79	B	0.0045630	0.060419515
VEQMSENRKG	BIP223_6/OSIP223_6	3PYA	A	0.0045880	0.060518657
QFTCNCMFIC	OSIP108_6	4IUT	B	0.0046500	0.060812072

Ligand	SIP	PDB_match	PDB Chain	Raw *p*-values (PepSite2)	BH correction (FDR=0.25)
NLEILEWIEI	BIP235_4/OSIP235_4	5GIJ	B	0.0046660	0.060891051
VMSVSPSVVW	BIP237_2/OSIP237_2	1WH2	A	0.0047450	0.061247208
LPLVSISSGA	BIP240_6/OSIP240_6	5ECK	A	0.0047450	0.061247208
RLTELGQRTT	BIP31_5	2FGE	B	0.0047450	0.061247208
SGLSNTALAR	BIP229_2/OSIP229_2	2Q3O	B	0.0047530	0.061280118
QQERNREFLK	BIP201_1/OSIP201_1	3T33	A	0.0047930	0.061451001
EAMISWLRV	BIP101_6/OSIP98_6	4XK8	7	0.0048620	0.06177667
FFFLHK	BIP91_2/OSIP88_2	4J0M	B	0.0048940	0.061934361
LLTSYELSCV	BIP231_3/OSIP231_3	4PYH	A	0.0049980	0.062359661
PVYRQMAQFT	BIP90_3	1T1H	A	0.0050570	0.062607232
HSEKDRLRKN	BIP40_1/OSIP44_1	2VTB	B	0.0051680	0.063083211
NTYLPIVSYR	BIP244_9/OSIP244_9	1XJ5	C	0.0051770	0.063126435
RIFNFPFRIK	BIP44_5/OSIP49_5	4QEO	A	0.0051770	0.063126435
PPPPPRFYVP	BIP161_4/OSIP147_4	5CTO	C	0.0053000	0.063655015
SSSIQSGCFF	BIP106_4	4NFU	B	0.0053980	0.064069027
PSPRYTSHSQ	BIP223_3/OSIP223_3	3H7R	A	0.0054710	0.064367463
ILTWFGQAKS	BIP226_1/OSIP226_1	4Z63	A	0.0055360	0.064652478
IQLMKYSFI	BIP244_9/OSIP244_9	2Q41	D	0.0055780	0.064836238
ISIVCFVCLK	BIP216_1/OSIP216_1	1U3C	A	0.0056770	0.065218571
LNYNVQ	BIP97_1	5E1J	A	0.0058630	0.065999611
IKIANFDKVK	BIP97_6	5KOP	B	0.0058800	0.066060605
RGEEQIFWCQ	BIP227_1/OSIP227_1	4O7G	A	0.0059100	0.066195474
RRREERSQRK	BIP210_2/OSIP210_2	4TNM	A	0.0059200	0.066236813
CGRGARVADM	OSIP86_3	5DQR	E	0.0059790	0.066476556
FFFFLY	BIP105_2	5FT9	B	0.0061030	0.066999648
NTLKHNITLE	BIP222_1/OSIP222_1	5KOR	A	0.0062250	0.067472018
NKLNPLSHSQ	BIP38_2/OSIP43_2	3CQR	A	0.0062350	0.067508689
NHMHKLE	BIP97_6	5KOP	C	0.0064900	0.06854416
HGTIIKKPNR	BIP242_7/OSIP242_7	3OGK	D	0.0065670	0.068828196
NFDKVKFQQH	BIP97_6	4QQR	B	0.0065670	0.068828196
PFLCRI	BIP202_1/OSIP202_2	1U1U	A	0.0065720	0.068874194
SWSKTL	BIP228_1/OSIP228_1	2G0Q	A	0.0065720	0.068874194
MMWQNMICYF	BIP227_1/OSIP227_1	4O7G	B	0.0066560	0.069168259
FITRKQSIED	BIP231_1/OSIP231_1	4TNM	A	0.0067230	0.069445878
IRPIRISVLY	BIP210_2/OSIP210_2	4TNM	A	0.0067340	0.069484908
IFQSYRLN	BIP223_3/OSIP223_3	3H7R	A	0.0067650	0.069622516
FSLTDFRAWL	BIP237_2/OSIP237_2	1WH2	A	0.0068940	0.070151807
LFWLYCPRLM	BIP233_2/OSIP233_2	4Z63	A	0.0069630	0.070419884
WHHKLERTSR	BIP122_1	4NC4	C	0.0070100	0.070601972
AAVERGTTPL	BIP97_1	5E1J	A	0.0073580	0.071991072
QKCNMYAKLS	BIP240_9/OSIP240_9	1U1U	A	0.0073830	0.072087261
CVKHRTTSIP	BIP89_4	5GZZ	D	0.0074330	0.072284489
SIELRRFRK	BIP89_6	5I32	A	0.0074360	0.072306175
VKCKLWKEVR	BIP155_6	4XK8	4	0.0075330	0.072680874
EYTDSIFPVL	BIP245_4/OSIP245_4	5D79	A	0.0075830	0.072871421
HKSASNNFFL	BIP105_5	4Z63	A	0.0077240	0.073404462

Ligand	SIP	PDB_match	PDB Chain	Raw *p*-values (PepSite2)	BH correction (FDR=0.25)
RSTDGVGRRG	BIP231_3/OSIP231_3	3NME	A	0.0077370	0.073453393
IHLYSGRDCW	OSIP68_6	3R0Q	G	0.0077500	0.073502431
SMFFFFLY	BIP105_2	3EI6	B	0.0079540	0.074310897
QYILSTHQHI	BIP230_5/OSIP230_5	2Q42	A	0.0080670	0.074711944
NHHRNRCQDR	BIP24_1	1RP0	A	0.0080940	0.07481054
VLVNANLDVE	BIP228_1/OSIP228_1	2G0Q	A	0.0081210	0.074908671
RSSEPSQCLV	BIP26_4/OSIP30_4	1YHY	A	0.0082030	0.075214494
TLLNKNNKAK	BIP216_1/OSIP216_1	1U3C	A	0.0083140	0.075642814
TFYLHLVFQ	BIP226_1/OSIP226_1	4Z63	A	0.0083440	0.075762288
SCTQVIVIEI	BIP209_2/OSIP209_2	3R0Q	C	0.0083830	0.075893327
ISVDVRFHLS	BIP209_2/OSIP209_2	3R0Q	G	0.0086100	0.076737689
REWMVTPFVK	BIP239_5/OSIP239_5	2CDQ	B	0.0087120	0.077115047
CMRRETINKR	BIP38_4/OSIP43_4	1XQ1	A	0.0087120	0.077115047
VLQVRNLPFL	BIP6_2/OSIP8_2	4LSA	A	0.0087550	0.077266844
VSCSAYKLYQ	OSIP71_2	3DM0	A	0.0087550	0.077266844
PEFSMDPSRL	OSIP114_5	3AX1	A	0.0088580	0.077649003
SRGLFIGRSC	BIP214_1/OSIP214_1	5K3S	A	0.0089180	0.077878839
AELVQGARLR	BIP180_1/OSIP167_1	5HAD	A	0.0089330	0.077933395
AELVQGARLR	BIP180_1/OSIP167_1	4MN8	A	0.0089330	0.077933395
RFALQNKLQR	BIP38_2/OSIP43_2	4MNA	A	0.0089480	0.07798838
ITQQQHIHTR	BIP231_4/OSIP231_4	4IH4	D	0.0089630	0.078050495
QTAPNHLKTT	BIP240_9/OSIP240_9	1U1J	A	0.0090380	0.078322818
TIHITRNKTL	BIP222_1/OSIP222_1	5KOP	B	0.0091290	0.0786594
LIMFTSSFLY	BIP10_6	5KOP	B	0.0092510	0.079105049
ESHQGSSNEI	BIP208_5/OSIP208_5	2Q3O	B	0.0093760	0.079560383
KIAFCLSFGV	BIP218_2/OSIP218_2	4NFU	B	0.0094540	0.079842866
SLISVGLTKN	BIP224_3/OSIP224_3	2G5W	B	0.0095180	0.080067059
PEIRFQVVLS	BIP231_4/OSIP231_4	4IH4	C	0.0097100	0.080740097
FNRLSFNGPK	BIP105_5	4Z63	A	0.0098730	0.081339058
VLETEARPFL	BIP214_1/OSIP214_1	5K2O	A	0.0100100	0.081859139
PQVVDIFPPG	BIP31_1	2YIJ	A	0.0100200	0.081864474
VCLNRQTKLV	BIP212_2/OSIP212_2	5D79	A	0.0100700	0.082032887
LTYGFPQVVL	BIP239_3/OSIP239_3	1U3D	A	0.0100900	0.082118636
RVEINEHLQS	BIP86_1	3PYB	A	0.0102300	0.082639422
DRRCFPCHPS	BIP24_1	5FDN	A	0.0102600	0.082723952
EIIVTLFLI	BIP231_2/OSIP231_2	3OGM	P	0.0104300	0.083333121
DSIPLGFRFL	BIP243_4/OSIP243_4	4J0M	A	0.0106600	0.084167306
PKTQFNIFPP	BIP0_3	4LSX	A	0.0107300	0.084410208
LVARLRLRAQ	BIP109_6/OSIP106_6	5FT9	B	0.0112800	0.086337975
FQFARKQLQI	BIP240_7/OSIP240_7	5HZG	F	0.0113000	0.086408625
LEKVVENTVA	BIP3_1	4BQE	A	0.0114100	0.086777945
SSRQFQSPNH	BIP6_2/OSIP8_2	4LSX	B	0.0114500	0.086918879
IAFLDLTILF	BIP239_3/OSIP239_3	1U3D	A	0.0114700	0.086990416
SLYSHVYCVI	BIP149_5/OSIP141_5	4LIX	A	0.0115100	0.087128803
KNGYLVSQVN	BIP86_6	4LIX	A	0.0116100	0.087468853
SCGHKSHIFC	BIP230_5/OSIP230_5	1XM8	A	0.0117200	0.087816072

Ligand	SIP	PDB_match	PDB Chain	Raw *p*-values (PepSite2)	BH correction (FDR=0.25)
SNRLTMTGSK	BIP154_1/OSIP145_1	4NC4	C	0.0117600	0.087958
IPYWSFVTSG	BIP51_6	2I9Y	A	0.0117600	0.087958
RVQYYKVYKA	OSIP77_2	1WE9	A	0.0118000	0.088099679
NPTQIFDIFG	BIP105_2	3EI8	B	0.0119000	0.088447917
LLSSCRSKAV	OSIP51_4	2FGE	A	0.0120000	0.088794146
NMSTLVQIQL	BIP91_2/OSIP88_2	4J0M	A	0.0121400	0.089260996
MTICLRSLPS	BIP245_4/OSIP245_4	5D79	B	0.0121600	0.089333243
SKRVLQSLKS	OSIP99_6	4NFU	B	0.0121800	0.089403622
ITCSITHYRR	BIP237_2/OSIP237_2	1WH2	A	0.0125700	0.090697647
RRLTGSYTRD	BIP34_4/OSIP39_4	1XM8	B	0.0127200	0.09122477
FWSERAWVQI	BIP180_1/OSIP167_1	5HAD	A	0.0128500	0.091677604
RKPLLQFTGE	BIP206_1/OSIP206_1	5FDN	A	0.0130000	0.092179449
DIPYIFLGAR	BIP214_1/OSIP214_1	1YI0	A	0.0131300	0.092612555
CTDILFWDAD	OSIP143_1	2ZFD	A	0.0133000	0.093166134
RFLNFQRINV	BIP239_2/OSIP239_2	1MVL	A	0.0133500	0.093347903
VKGEVTETKE	BIP155_6	4XK8	9	0.0135000	0.09382991
VQRIFNFPFR	BIP44_5/OSIP49_5	4XAE	A	0.0135500	0.094011457
NRQTKLVFAV	BIP212_2/OSIP212_2	2EVN	A	0.0135900	0.094124585
THSDIEMTSL	BIP232_2/OSIP232_2	5KSD	B	0.0136400	0.09429913
WTHSQEPGTP	OSIP36_4	4OUR	B	0.0137300	0.094600892
TFEFFSSSLI	BIP227_1/OSIP227_1	4O6Y	A	0.0138200	0.094887131
VLACLGYSHY	BIP149_5/OSIP141_5	4LIX	A	0.0138700	0.095056799
IYCRSWRAIV	OSIP86_3	5DQR	A	0.0138700	0.095056799
RDSQLINCPF	BIP239_2/OSIP239_2	1MVL	A	0.0138900	0.095116156
WAKISTVLEI	BIP231_2/OSIP231_2	2P1N	B	0.0140300	0.095584402
LGSCLVLLIL	BIP3_1	4BQE	A	0.0140800	0.095755287
GPVPPSQPSP	BIP38_2/OSIP43_2	4MNA	A	0.0141000	0.09580823
GFMSYYVLL	BIP10_6	5KOP	B	0.0142600	0.096312321
RMCQPDTTSY	BIP206_1/OSIP206_1	5FDN	A	0.0143400	0.09658743
QIPSLSEEMP	BIP164_2	4Z63	A	0.0143800	0.096690656
SQYSGLHSRS	BIP62_3/OSIP58_3	4RL5	B	0.0145800	0.097357486
GPIISCLHNH	BIP91_2/OSIP88_2	4J0M	A	0.0146200	0.097463191
SLEIAVKSVM	BIP175_4	1U3C	A	0.0147500	0.097887324
AAYWYEWRRK	BIP200_2/OSIP200_2	3HTX	A	0.0147700	0.097936545
KISSQGTFSH	BIP236_3/OSIP236_3	4N7Q	A	0.0148700	0.098253494
GFRSENTCRL	BIP218_2/OSIP218_2	4NFU	B	0.0149400	0.098469404
LILSHLQALP	BIP90_3	1T1H	A	0.0157600	0.101087414
SASNNFFLLI	BIP105_5	2Q4W	A	0.0161000	0.102139351
LLNLVRNSCF	BIP218_2/OSIP218_2	4NFU	B	0.0161000	0.102139351
LPQSSIVDPS	BIP223_3/OSIP223_3	3H7R	A	0.0167300	0.104092779
LVQNTKGKLT	BIP174_1	2P1N	D	0.0170100	0.104949204
QSILVRIHLV	BIP233_2/OSIP233_2	4Z63	A	0.0170100	0.104949204
IFLMFNFCVY	OSIP99_3	4Z61	B	0.0170600	0.105087571
WALIGLFKDL	BIP105_5	4Z63	A	0.0173500	0.106009891
FFNGYSLLWL	BIP34_4/OSIP39_4	2Q42	A	0.0175200	0.106513215
TRLQSGVRVF	BIP208_2/OSIP208_2	4GL4	A	0.0179900	0.107922622

Ligand	SIP	PDB_match	PDB Chain	Raw *p*-values (PepSite2)	BH correction (FDR=0.25)
CYSFFNC	BIP206_2/OSIP206_2	1U1U	A	0.0181700	0.108459676
LFTEVRITKH	BIP235_1/OSIP235_1	3H7R	A	0.0181700	0.108459676
KQNSNKKEVK	BIP243_3/OSIP243_3	5HYX	B	0.0182300	0.10864121
KLEWDNVWKS	BIP86_1	4LIX	A	0.0182900	0.108813346
QNLFCNLSDA	BIP63_1	4Z61	B	0.0183200	0.108898748
LKIRMEKNDD	BIP242_7/OSIP242_7	3OGK	F	0.0184700	0.109349234
DEYDCWWSS	OSIP94_4	2HJ3	A	0.0185200	0.109519237
VKSPVPSRTK	BIP44_5/OSIP49_5	4XAE	A	0.0187800	0.110280281
KSWAKISTVL	BIP231_2/OSIP231_2	3OGM	H	0.0189700	0.110849915
STEVGLVLGR	BIP239_3/OSIP239_3	1U3D	A	0.0190000	0.110940584
ETYGTLCI	BIP90_4	3DM0	A	0.0190300	0.111025685
RQGVPQTERR	BIP67_2/OSIP64_2	3HBX	C	0.0191600	0.111408625
HSRSNHNTAP	BIP62_3/OSIP58_3	4Z61	B	0.0192500	0.111664391
QSFACLASDM	BIP142_3/OSIP134_3	4LSX	B	0.0195100	0.112443313
LDSLSRCKLR	BIP90_4	3DM0	A	0.0201300	0.114259638
VALFHVHAVN	BIP171_2/OSIP158_2	1YDW	A	0.0201600	0.114336491
ANIFFLVVTN	BIP234_1/OSIP234_1	5FT9	B	0.0203000	0.11476147
CHNKLNPLSH	BIP38_2/OSIP43_2	4MNA	A	0.0203600	0.114932168
CCFGLIGKKR	BIP240_5/OSIP240_5	2NTY	B	0.0206700	0.115850134
RRRGLGSKGD	BIP171_4/OSIP158_4	5E1J	A	0.0207400	0.116056591
FHLLAGLVWL	OSIP99_1	2Q4E	A	0.0213300	0.117772765
CAEPQRGRC	BIP51_6	2I9Y	A	0.0213400	0.117825734
SAEVPPWIPC	BIP90_3	1T1H	A	0.0213600	0.117844351
PISTIDSVNE	BIP22_1/OSIP25_1	3OGK	N	0.0214300	0.11804765
FLHNHKSYSH	BIP242_2/OSIP242_2	1N7G	C	0.0221500	0.120075806
RQFKALYQYI	BIP4_2/OSIP5_2	5HH7	A	0.0226700	0.121560274
LKYIKMEHNN	BIP243_3/OSIP243_3	5HYX	B	0.0228900	0.122170217
LDSSTSHVGK	OSIP99_6	4NFU	B	0.0229700	0.122399955
VQLTTES	BIP62_3/OSIP58_3	4RL5	B	0.0230800	0.122691886
RVCLLVLARF	BIP240_7/OSIP240_7	5HZG	B	0.0231200	0.122801725
RSMLQRVRLV	BIP88_4/OSIP84_4	5HTR	A	0.0232300	0.123108674
EKFSPED	BIP212_3/OSIP212_3	5FDN	A	0.0236900	0.124444471
KRTIKNTYIG	BIP240_4/OSIP240_4	4CXV	A	0.0238500	0.124846516
NNRESERGEN	BIP240_5/OSIP240_5	2NTY	B	0.0239700	0.125180233
ILERRIYPSV	BIP233_2/OSIP233_2	4Z63	A	0.0240500	0.125412996
AFNRSRRALR	BIP224_2/OSIP224_2	2VY2	A	0.0242100	0.125866341
SQVYSSHTYV	BIP38_2/OSIP43_2	4MN8	A	0.0242900	0.126083567
RGRYTFLHTY	OSIP94_4	2HJ3	A	0.0244500	0.126522559
EQRLGFHLLA	OSIP99_1	5GQ0	B	0.0246100	0.126958916
LQIAVCFANL	BIP212_3/OSIP212_3	5FDN	A	0.0246900	0.127175734
DLNQPKMYKF	BIP229_2/OSIP229_2	2XCM	F	0.0248100	0.127498098
LVTGFTS	OSIP114_5	3AX1	A	0.0248200	0.127543025
ALNNYRLQIP	OSIP103_6	2C59	A	0.0253500	0.128950803
VLLVVTYHRE	BIP236_3/OSIP236_3	4N7Q	A	0.0254300	0.129157373
VQEKVITSSL	OSIP50_2	5A5K	S	0.0254300	0.129157373
RIWSLQLKYT	BIP235_1/OSIP235_1	3H7R	A	0.0255100	0.129363564

Ligand	SIP	PDB_match	PDB Chain	Raw *p*-values (PepSite2)	BH correction (FDR=0.25)
FAQSYSFFLQ	BIP50_3	2J3H	A	0.0256400	0.129731668
KKELIIFFSG	OSIP99_6	4NFU	B	0.0257200	0.129936132
RQNHHRNRCQ	BIP24_1	5FDN	A	0.0258500	0.130300278
MMNSIDNKNQ	BIP243_4/OSIP243_4	4J0M	A	0.0258500	0.130300278
KTGDAYLSSG	BIP34_4/OSIP39_4	1XM8	B	0.0261500	0.131109139
GTNFVTRTLV	BIP208_6/OSIP208_6	1N7G	C	0.0262300	0.131318332
KKLQDDAMMA	BIP226_2/OSIP226_2	4FRZ	A	0.0262300	0.131318332
FGGVESWIHI	BIP38_4/OSIP43_4	1XQ1	A	0.0263200	0.131568994
IVIDRVQICN	BIP218_3/OSIP218_3	5TEB	H	0.0265400	0.132158608
SIHHRFESSF	BIP240_4/OSIP240_4	4CWM	B	0.0266700	0.132496868
NNGDVREDLS	BIP86_1	3PYA	A	0.0269800	0.133347274
VFSYFMDNKR	BIP224_2/OSIP224_2	2VY2	A	0.0270200	0.133442025
IPMLYMLVSY	BIP109_2/OSIP106_2	1U1H	A	0.0272900	0.134157036
SSVFVYLLQV	BIP89_2	4EQ4	B	0.0277400	0.135346468
RRYRFPNSCL	BIP226_4/OSIP226_4	5HTR	A	0.0282900	0.136803225
PTRVFGNRVS	BIP233_1/OSIP233_1	3RIZ	A	0.0282900	0.136803225
DFDCRNNCGV	BIP97_3	3PPZ	A	0.0284700	0.137259041
LDIRFMPERY	OSIP52_5	2WTB	A	0.0284700	0.137259041
QSFSVQLTTE	BIP62_3/OSIP58_3	4Z61	A	0.0288000	0.138130201
SCDNSQYSGL	BIP62_3/OSIP58_3	4Z61	A	0.0290400	0.138766252
DCAENEYDRN	OSIP70_3	2Q45	A	0.0290400	0.138766252
FVSNCFIWKG	BIP217_2/OSIP217_2	4Z61	B	0.0291800	0.139124059
MMQVISEVVS	BIP206_1/OSIP206_1	5FDN	A	0.0293700	0.139612889
KEVHDGRVMV	BIP101_6/OSIP98_6	4XK8	7	0.0298100	0.14076609
HVHKFCEACS	BIP218_3/OSIP218_3	5TEB	F	0.0299100	0.141022794
MYIHEIEMKK	BIP34_4/OSIP39_4	2Q42	B	0.0299100	0.141022794
IFLFFWFHAL	BIP244_1/OSIP244_1	4N0G	D	0.0299500	0.141114425
IHTHMDLIIS	BIP202_1/OSIP202_2	1U1U	A	0.0301500	0.141621516
DPDNPTQIFD	BIP105_2	2APJ	B	0.0302000	0.141754676
LKHLPIPRHK	OSIP103_6	2C5E	B	0.0302000	0.141754676
DILFWDADFP	OSIP143_1	2P1N	D	0.0304000	0.142277991
VIENQVRFDC	BIP174_1	2P1N	D	0.0305000	0.142532498
TGNSSTLVSS	OSIP36_5	2WSF	A	0.0306000	0.142784996
NLCQAYIVLH	BIP124_5/OSIP115_5	5FDN	A	0.0310000	0.143811587
GQLCPSLNWG	BIP31_1	5FDN	A	0.0310000	0.143811587
QLSTYQKPNR	BIP142_4/OSIP134_4	4N0G	A	0.0313500	0.144748439
QNPYQIKKNL	BIP218_1/OSIP218_1	2EFC	C	0.0315100	0.145140998
SKESISISVS	BIP89_6	5I32	A	0.0317100	0.145640736
LFQVVTALVC	BIP212_3/OSIP212_3	5FDN	A	0.0319200	0.146170456
QVAYWRLHSK	BIP43_1	2DGE	D	0.0319700	0.146283498
HTTWWSSMSS	BIP240_9/OSIP240_9	1U1U	A	0.0321800	0.146809666
SLLPFSLTDF	BIP237_2/OSIP237_2	5IGO	A	0.0322900	0.147101005
YRFYFLII	OSIP114_6	1GCC	A	0.0331500	0.149282028
GLVWLVNPSR	OSIP99_1	5GQ0	B	0.0332000	0.149393423
DMSIICMFSV	BIP240_4/OSIP240_4	4CXV	A	0.0332500	0.149525729
RVSRRRKSSI	OSIP51_4	2FGE	B	0.0334100	0.149925544

Ligand	SIP	PDB_match	PDB Chain	Raw *p*-values (PepSite2)	BH correction (FDR=0.25)
NQILEQHDPI	BIP214_1/OSIP214_1	4XK8	H	0.0335800	0.150348193
SVITYPSCTQ	BIP209_2/OSIP209_2	2ZFD	A	0.0338500	0.151003875
RLQHHAESLP	BIP208_6/OSIP208_6	1N7G	B	0.0340700	0.151535625
TLVATPRCVS	BIP97_6	4QQR	B	0.0346900	0.153090811
AVMEKQSKVC	BIP109_2/OSIP106_2	1U22	A	0.0350300	0.153920874
LNHHCRFSGK	BIP243_2/OSIP243_2	5E4W	D	0.0352500	0.154436627
VATRQSPRVP	BIP122_2	3VEM	C	0.0353100	0.154590507
LGNQRKKAYH	BIP212_3/OSIP212_3	5FDN	A	0.0353700	0.154744637
SGKINLQKAQ	BIP66_2	5DQQ	A	0.0353700	0.154744637
NLPAIFVSQK	OSIP71_2	3DM0	A	0.0354800	0.155000615
RHFLEQSRLP	BIP89_5	5JO2	B	0.0361800	0.156730397
CKYAMQCNAK	BIP209_1/OSIP209_1	4LDY	A	0.0367100	0.158007259
HMIMASCDFV	BIP97_3	3PPZ	A	0.0367100	0.158007259
EHENEITVCL	BIP212_2/OSIP212_2	2Q4Y	A	0.0367700	0.158154087
LCHVYSAVRC	BIP233_1/OSIP233_1	4M7E	B	0.0372500	0.159294505
VEGSTISYCC	OSIP51_4	2FGE	A	0.0373700	0.159574315
SQRIMPLVIA	BIP208_2/OSIP208_2	1JXC	A	0.0375600	0.160035916
LMGTDCKFVG	BIP91_2/OSIP88_2	4J0M	A	0.0382300	0.161646753
RFFQSMIAAS	BIP218_4/OSIP218_4	4QNK	A	0.0382900	0.161784431
RRQIADIMI	BIP67_2/OSIP64_2	3HBX	F	0.0384800	0.162235883
STDYGFNFFF	BIP66_1	4EQ4	A	0.0385400	0.162367483
EMDSVLGGSE	OSIP70_3	2Q45	A	0.0389200	0.163275279
EPTIVPDCLS	BIP235_4/OSIP235_4	2Q4W	A	0.0390400	0.16356266
SRWSLEIAVK	BIP175_4	2CF6	A	0.0392300	0.164012724
MFLGDRIMFF	BIP10_6	2Z20	A	0.0394200	0.164455755
LPQVVDIFPP	BIP31_1	5FDN	A	0.0394900	0.164618986
MKKEDIGRRF	BIP38_2/OSIP43_2	3CQR	B	0.0394900	0.164618986
ILKSIRHMRY	BIP243_2/OSIP243_2	5E4W	D	0.0395500	0.164761219
NDLSKEVAPG	BIP31_1	4EQ4	B	0.0395500	0.164761219
HSDLQKDSWV	BIP90_3	1T1H	A	0.0402000	0.166266114
TSITLPMR	BIP4_2/OSIP5_2	5HH7	A	0.0402700	0.166425248
EELARLDKFD	OSIP71_2	3DM0	A	0.0403900	0.166689884
EVERQENGWF	BIP175_4	2CF5	A	0.0404600	0.166860488
YLSKFLISHF	BIP242_2/OSIP242_2	1N7G	B	0.0407800	0.167589395
KKNEKEKKFM	BIP240_5/OSIP240_5	2WBL	A	0.0410500	0.168220181
EQRIKQNK	BIP224_4/OSIP224_4	4RQT	A	0.0410700	0.168295543
VLLPNSFFPL	OSIP99_3	4Z61	B	0.0411800	0.168515404
KITRGHNHMH	BIP97_6	4N7R	C	0.0412500	0.168685191
QLCPSLNWGT	BIP31_1	2YIJ	A	0.0415100	0.169276762
SSPPSLLSQA	BIP242_6/OSIP242_6	1TG5	A	0.0417800	0.169899522
RAWLVMSVSP	BIP237_2/OSIP237_2	5IGO	B	0.0419200	0.170215209
QAVKKLRID	BIP71_4/OSIP67_4	4RQW	A	0.0421900	0.170817745
CEKERKWTMN	BIP209_1/OSIP209_1	4LDY	A	0.0422600	0.170982385
PKSNRFQFAL	BIP238_2/OSIP238_2	1ZWJ	A	0.0426000	0.171753791
FTNKQKKFIQ	BIP218_1/OSIP218_1	2EFD	C	0.0426700	0.17190842
SSPKIPDSPV	BIP209_1/OSIP209_1	4EET	B	0.0430100	0.172679142

Ligand	SIP	PDB_match	PDB Chain	Raw *p*-values (PepSite2)	BH correction (FDR=0.25)
WRNLKEMRQR	BIP142_2/OSIP134_2	5CTO	C	0.0435700	0.173960074
IWFRINNELE	BIP243_4/OSIP243_4	4J0M	B	0.0444200	0.175876905
PVLQRKPKLV	BIP37_5	3UDB	E	0.0447100	0.176507265
VSTRYNILCY	BIP206_2/OSIP206_2	1W07	A	0.0447800	0.176655911
VRSRREGKSK	BIP3_1	4BQE	A	0.0450700	0.177310043
VGILIDLCH	BIP73_2/OSIP75_2	4MN8	B	0.0453500	0.177952663
SNLLYLATVT	BIP239_2/OSIP239_2	1MVL	A	0.0453600	0.177952663
VSFITVSYHF	BIP240_4/OSIP240_4	4CWM	B	0.0455800	0.178423641
KSVRAGSNYI	BIP242_2/OSIP242_2	1N7G	B	0.0458700	0.179047364
SPVPSRTKFR	BIP44_5/OSIP49_5	4QEO	A	0.0463900	0.180176282
TRCITCHLRS	BIP106_6	4Q75	B	0.0464600	0.180324403
QHLDDKLFVR	BIP226_2/OSIP226_2	4FRZ	A	0.0464600	0.180324403
GTARRRSLEL	BIP224_2/OSIP224_2	2VY2	A	0.0465400	0.180504204
KIGHHCRRRG	BIP219_3/OSIP219_3	4IC6	B	0.0466100	0.18065348
PRYTSHSQIF	BIP223_3/OSIP223_3	4QQR	B	0.0471400	0.181800966
TQKKGEVNLV	BIP243_3/OSIP243_3	5HYX	B	0.0474400	0.182452564
QEQQGQDLHR	BIP216_2/OSIP216_2	2CDQ	B	0.0479000	0.183444151
RLSFNGPKWA	BIP105_5	2Q4W	A	0.0481300	0.183930817
MTLCFNAVNT	BIP5_1/OSIP6_1	2EFD	A	0.0487500	0.185272361
LSIHSTKNRR	BIP6_2/OSIP8_2	4LSA	A	0.0487500	0.185272361
CYHGVSYTHS	BIP129_6	5D79	B	0.0489100	0.18561521
NPKSNRFQFA	BIP238_2/OSIP238_2	4NFU	A	0.0490600	0.185925994
TYMYPRFDSS	BIP38_1/OSIP43_1	5HYW	A	0.0491400	0.1860935
IIIIDRNPQS	BIP225_2/OSIP225_2	4NT1	D	0.0492200	0.186264308
IILEHLVLLM	BIP233_2/OSIP233_2	4Z63	A	0.0493000	0.18643251
FVILTSVNNV	BIP235_2/OSIP235_2	4EQ4	B	0.0493000	0.18643251
KHSKCKYAMQ	BIP209_1/OSIP209_1	4EET	B	0.0493800	0.186600817
VMGPFSGPSE	OSIP168_1	4EQ4	A	0.0496900	0.187261714
VFRMSENPSE	BIP216_2/OSIP216_2	2CDQ	B	0.0499300	0.18778169
IFGSMFFFFL	BIP105_2	1PXY	B	0.0504100	0.188806995
KTTFNISGQE	BIP0_3	3RIZ	A	0.0507400	0.189512531
SPLFVKMLTR	OSIP24_6	4NFU	A	0.0507400	0.189512531
KLWILPLIIS	BIP89_2	4FYP	B	0.0509800	0.190011935
EDVSTRYNIL	BIP206_2/OSIP206_2	1U1J	A	0.0514700	0.1910408
SHRRRIRRLC	BIP31_1	5FDN	B	0.0517200	0.191553202
ICEHQTPIHS	BIP4_2/OSIP5_2	5HH7	A	0.0518800	0.191872228
LFIIVLMRLV	BIP216_2/OSIP216_2	2CDQ	B	0.0522200	0.192586714
FLARNVECRF	BIP0_6	4XK8	G	0.0535600	0.195366277
QPSALIPCWI	BIP230_2/OSIP230_2	1IBJ	A	0.0536500	0.195558319
PNLVVFNGSR	OSIP114_6	1GCC	A	0.0536500	0.195558319
DPEKRDPDNP	BIP105_2	5FT9	B	0.0541600	0.196616989
FDIKKIFRWI	BIP204_2/OSIP204_2	5HTR	A	0.0546000	0.197528662
IIPMLKHIIE	BIP88_3/OSIP84_3	2VY2	A	0.0549400	0.19822637
QGAFGRWSRV	BIP155_5	5L25	A	0.0554700	0.199300184
FSLNCFGCYV	BIP73_2/OSIP75_2	4MN8	B	0.0561800	0.200728631
RKRRNNFSCS	BIP142_4/OSIP134_4	4N0G	B	0.0567200	0.201827368

Ligand	SIP	PDB_match	PDB Chain	Raw *p*-values (PepSite2)	BH correction (FDR=0.25)
LTQETLRPYF	BIP218_1/OSIP218_1	2EFD	A	0.0567200	0.201827368
SHSQIFQSYR	BIP223_3/OSIP223_3	3UC3	A	0.0570800	0.202558839
GSGSFLPICS	BIP240_9/OSIP240_9	1U1U	A	0.0571700	0.202747044
YYQPKEIRPL	OSIP52_5	2WTB	A	0.0573500	0.2030957
REESQQTKWV	BIP210_3/OSIP210_3	2VCE	A	0.0574400	0.203277565
TSKKDSVFLL	BIP6_2/OSIP8_2	3RIZ	A	0.0577100	0.203811759
QKNGQKENKV	BIP221_3/OSIP221_3	3NMV	B	0.0580800	0.204537751
LANQHLQKAN	BIP21_5/OSIP23_5	2EFD	A	0.0581700	0.204708051
VNGFSLNLSK	OSIP114_6	1GCC	A	0.0584500	0.205260426
VCFHKDINLI	BIP243_2/OSIP243_2	5E4W	C	0.0591900	0.206738965
QEEKKTTLRE	BIP71_4/OSIP67_4	4RQW	A	0.0592900	0.206938718
NLLPSFFIIF	BIP168_4/OSIP154_4	2O01	H	0.0594700	0.207294513
RECLSQRIMP	BIP208_2/OSIP208_2	4GQY	C	0.0598500	0.208028198
FQSGRFVCGD	BIP233_1/OSIP233_1	4M7E	A	0.0598500	0.208028198
CSQLSTYQKP	BIP142_4/OSIP134_4	5A4X	A	0.0605200	0.209360044
WRENVKLCFW	BIP0_6	4XK8	G	0.0607100	0.209736658
HSSDPVNIWK	BIP235_4/OSIP235_4	2Q4W	A	0.0607100	0.209736658
FMQFGDRQIR	BIP59_1	3UI2	A	0.0608000	0.2098965
ALRNTSGRIP	BIP68_1	4OH3	B	0.0610000	0.210303386
IFALQKLKSL	BIP243_3/OSIP243_3	5HYX	B	0.0611900	0.210671458
LLNIFWFDQY	BIP238_2/OSIP238_2	4NFU	A	0.0613800	0.211030445
KSSLRHWSQS	BIP233_1/OSIP233_1	4M7E	B	0.0616700	0.211599594
FTKVFIYNTK	BIP69_2/OSIP65_2	2EFD	C	0.0627500	0.213696293
SVVWITCSIT	BIP237_2/OSIP237_2	5IGO	A	0.0632500	0.21464167
FPLDFHRVMM	BIP208_5/OSIP208_5	2Q4L	B	0.0633500	0.21482719
YFIPICVVTP	BIP245_1/OSIP245_1	5KOD	B	0.0636500	0.215404009
QIRQRSDKKS	BIP59_1	3UI2	A	0.0639500	0.21597445
QIRQRSDKKS	BIP59_1	3DEO	A	0.0639500	0.21597445
RKNVEYHFWR	BIP0_6	4XK8	G	0.0641500	0.216354499
PFLKRIEIDS	BIP238_3/OSIP238_3	1VK0	A	0.0647600	0.217503744
IKEEVFEICI	BIP223_4/OSIP223_4	2RU1	A	0.0650600	0.218048303
NQARGAEEIV	BIP37_5	3UC3	A	0.0652700	0.218447687
VDPSPSPRYT	BIP223_3/OSIP223_3	3UC3	A	0.0659900	0.219818012
KKRNLDIIRA	BIP235_1/OSIP235_1	3H7R	A	0.0659900	0.219818012
MLLSHLFASL	BIP50_3	2J3I	B	0.0664000	0.220555451
FGGKMLNCVF	BIP0_5	4TJX	A	0.0666100	0.220953314
REEEHENEIT	BIP212_2/OSIP212_2	5D79	A	0.0673500	0.222331711
PIVQEKVITS	OSIP50_2	1YYC	A	0.0676600	0.222887975
LFFDDREKSS	BIP226_2/OSIP226_2	4FRZ	A	0.0680900	0.223714138
NFFAFFNPYG	BIP245_2/OSIP245_2	4PXB	A	0.0686200	0.224675848
RRYVIQLMKY	BIP244_9/OSIP244_9	2GM3	B	0.0687300	0.224884558
CMYGCMHECT	OSIP143_1	2P1N	D	0.0699200	0.227053587
KRIRDNILRR	BIP231_6/OSIP231_6	2JKI	B	0.0703600	0.227844047
KLKIPNTNLN	BIP238_2/OSIP238_2	2Q4L	B	0.0705800	0.228259968
NLDIKIIRRS	OSIP99_3	4Z61	B	0.0714600	0.229860994
IARPRIMIGE	BIP88_2/OSIP84_2	1OGP	F	0.0715800	0.230078928

Ligand	SIP	PDB_match	PDB Chain	Raw *p*-values (PepSite2)	BH correction (FDR=0.25)
HRCTSKIKVN	BIP24_1	5FDN	A	0.0718000	0.230472044
VVFGGKMLNC	BIP0_5	4TJV	A	0.0721400	0.231072809
EDRTVMLSFP	BIP216_3/OSIP216_3	1TIZ	A	0.0722500	0.231278692
AGKLNPFLMF	BIP6_6/OSIP8_6	1ZWJ	B	0.0723600	0.231469126
RTSGIIPMLK	BIP88_3/OSIP84_3	4N7R	C	0.0733800	0.233241367
VKYGAGCTRP	BIP240_7/OSIP240_7	1U1H	A	0.0737200	0.233837703
PQSSIVDPSP	BIP223_3/OSIP223_3	5E4W	B	0.0739500	0.23426728
SPSWRSTLPQ	BIP88_4/OSIP84_4	5HTR	A	0.0739500	0.23426728
MKRMAFCAKN	OSIP166_6	3UDB	E	0.0751100	0.236276555
ILVLLGRYCT	OSIP50_1	4QQR	B	0.0755800	0.237076815
GVQYILSTHQ	BIP230_5/OSIP230_5	3UDB	C	0.0764000	0.238478004
ENMQIIDISP	BIP240_6/OSIP240_6	5A3V	A	0.0766400	0.238906914
MNWRVDVKSS	BIP71_4/OSIP67_4	4RQW	A	0.0770000	0.239538762
VFYGSFSLTC	OSIP114_6	1GCC	A	0.0772300	0.239911326
CGILKEVHKN	BIP161_3/OSIP147_3	4FRZ	A	0.0774700	0.240315099
LEQSRLPPVE	BIP89_5	2VCH	A	0.0774700	0.240315099
NVFFGGKMLN	OSIP10_5	2J3I	B	0.0775900	0.240524458
RRNGRSLDWR	BIP224_2/OSIP224_2	2VY2	A	0.0782000	0.241573114
TNPQHNYGSG	BIP224_5/OSIP224_5	4XK8	6	0.0785600	0.242174961
FGHKLYNSLC	BIP38_1/OSIP43_1	5HZG	F	0.0791700	0.243211136
TPERSDPEKR	BIP105_2	2APJ	B	0.0794100	0.243628722
HQNQIKEYFI	BIP244_8/OSIP244_8	4HHD	B	0.0796600	0.244062067
TSYFCRDNQG	BIP230_2/OSIP230_2	1IBJ	A	0.0816500	0.247415197
RSKSMNHQIY	BIP22_3/OSIP25_3	3B5I	A	0.0820300	0.248037959
