# Supplementary material for: Large-scale docking predicts that sORF-encoded peptides may function through protein-peptide interactions in Arabidopsis thaliana
Source: PLoS One. 2018 Oct 15;13(10):e0205179. doi: 10.1371/journal.pone.0205179 (PMC6188750; doi:10.1371/journal.pone.0205179)
Supplement: S2 Table — The table includes the PepSite2 p-values and ΔGbind values of the models. (PDF) [file pone.0205179.s006.pdf]

Ligand	SIP	PDB_match	PDB Chain	Raw *p*-values (PepSite2)	BH correction (FDR=0.25)	Average △G (kcal/mol)	Std. Dev.	Std. Err. of Mean
AAYWYEWRRK	BIP200_2/OSIP200_2	3HTX	A	0.014770	0.097936545	-48.35930	4.77940	0.4779
ALNNYRLQIP	OSIP103_6	2C59	A	0.025350	0.128950803	-8.39420	3.12150	0.3122
ALRIPKLYLV	OSIP85_3	2WSE	B	0.000253	0.043062533	-45.26180	5.13750	0.5137
APCPSLLHKL	BIP209_3/OSIP209_3	5IYX	A	0.000460	0.043062533	-0.63730	6.02520	0.6025
DEYDCWWSS	OSIP94_4	2HJ3	A	0.018520	0.109519237	-37.86870	4.12040	0.4120
DLNQPKMYKF	BIP229_2/OSIP229_2	2XCM	F	0.024810	0.127498098	-19.53060	3.84570	0.3846
EDVSTRYNIL	BIP206_2/OSIP206_2	1U1J	A	0.051470	0.191040800	-26.03370	6.33560	0.6336
EHENEITVCL	BIP212_2/OSIP212_2	2Q4Y	A	0.036770	0.158154087	-36.20320	5.51610	0.5516
EPTIVPDCLS	BIP235_4/OSIP235_4	2Q4W	A	0.039040	0.163562660	-13.90500	3.72670	0.3727
EQRIKQNK	BIP224_4/OSIP224_4	4RQT	A	0.041070	0.168295543	-15.58430	3.53910	0.3539
FAVEFQVRK	BIP212_2/OSIP212_2	5D79	B	0.004563	0.060419515	-49.89540	6.65090	0.6651
FFDDVKSRRL	BIP103_3	2XQR	A	0.004392	0.059654394	-29.55310	5.49970	0.5500
FGHKLYNSLC	BIP38_1/OSIP43_1	5HZG	F	0.079170	0.243211136	-34.48440	8.72100	0.8721
FLARNVECRF	BIP0_6	4XK8	G	0.053560	0.195366277	-22.16490	3.10120	0.3101
FLMFSYFWCC	BIP6_6/OSIP8_6	1U3D	A	0.004255	0.059077753	-34.50010	5.27970	0.5280
FSLTDFRAWL	BIP237_2/OSIP237_2	1WH2	A	0.006894	0.070151807	-15.47240	7.09220	0.7092
FTKVFIYNTK	BIP69_2/OSIP65_2	2EFD	C	0.062750	0.213696293	-20.12880	7.08030	0.7080
GIYLYNKCAF	OSIP68_6	3R0Q	G	0.003906	0.057508502	-31.84320	5.80910	0.5809
GLVWLVNPSR	OSIP99_1	5GQ0	B	0.033200	0.149393423	-1.90370	3.68130	0.3681
HQNQIKEYFI	BIP244_8/OSIP244_8	4HHD	B	0.079660	0.244062067	-11.40990	4.36070	0.4361
IARPRIMIGE	BIP88_2/OSIP84_2	1OGP	F	0.071580	0.230078928	-38.33590	5.86950	0.5869
IFALQKLKSL	BIP243_3/OSIP243_3	5HYX	B	0.061190	0.210671458	-0.91440	4.75250	0.4752
IFLFFWFHAL	BIP244_1/OSIP244_1	4N0G	D	0.029950	0.141114425	-39.59630	5.01380	0.5014
IIIIDRNPQS	BIP225_2/OSIP225_2	4NT1	D	0.049220	0.186264308	-10.35640	6.69850	0.6698
ILRRICILIT	BIP231_6/OSIP231_6	4A0G	A	0.002503	0.050945665	-50.44350	7.70320	0.7703
IPYWSFVTSG	BIP51_6	2I9Y	A	0.011760	0.087958000	-17.98000	6.68070	0.6681
ISCGHKSHIF	BIP230_5/OSIP230_5	5ECK	A	0.000815	0.043167944	-5.77180	4.34800	0.4348
ITQQQHIHTR	BIP231_4/OSIP231_4	4IH4	D	0.008963	0.078050495	-10.12720	3.73150	0.3732
KDLTFFPPKN	BIP0_3	3RIZ	A	0.002727	0.052016522	-17.44970	4.17330	0.4173
KELQNTLTYI	BIP201_1/OSIP201_1	3T33	A	0.004482	0.060052748	-20.93610	7.26090	0.7261
KEVHDGRVMV	BIP101_6/OSIP98_6	4XK8	7	0.029810	0.140766090	-29.43300	6.74470	0.6745

Ligand	SIP	PDB_match	PDB Chain	Raw *p*-values (PepSite2)	BH correction (FDR=0.25)	Average △G (kcal/mol)	Std. Dev.	Std. Err. of Mean
KGILTVQSAG	OSIP164_1	4KA8	A	0.000574	0.043062533	-40.50060	8.27320	0.8273
KHSKCKYAMQ	BIP209_1/OSIP209_1	4EET	B	0.049380	0.186600817	-14.28230	3.64740	0.3647
KIAFCLSFGV	BIP218_2/OSIP218_2	4NFU	B	0.009454	0.079842866	-7.04160	3.33890	0.3339
KRIRDNILRR	BIP231_6/OSIP231_6	2JKI	B	0.070360	0.227844047	-27.26120	6.73430	0.6734
KTGDAYLSSG	BIP34_4/OSIP39_4	1XM8	B	0.026150	0.131109139	-46.63500	4.82680	0.4827
LAEDTFGEIS	BIP142_3/OSIP134_3	2VTB	D	0.001674	0.046760405	-48.01700	6.07070	0.6071
LDIRFMPERY	OSIP52_5	2WTB	A	0.028470	0.137259041	-33.26080	5.68960	0.5690
LDRISKAQSV	BIP139_6	4PUT	A	0.000609	0.043062533	-59.79300	8.22630	0.8226
LDSLSRCKLR	BIP90_4	3DM0	A	0.020130	0.114259638	-5.47730	6.15430	0.6154
LEKVVENTVA	BIP3_1	4BQE	A	0.011410	0.086777945	-27.05020	6.28010	0.6280
LLTSYELSCV	BIP231_3/OSIP231_3	4PYH	A	0.004998	0.062359661	-15.79290	3.82530	0.3825
LNHHCRFSGK	BIP243_2/OSIP243_2	5E4W	D	0.035250	0.154436627	-10.30840	3.00680	0.3007
LNKICIFFST	BIP154_5/OSIP145_5	2O01	A	0.000976	0.043653769	-38.41440	4.16640	0.4166
LRSMAACFAY	BIP89_6	5I32	A	0.002063	0.048706951	-21.79080	3.72030	0.3720
LSQRIMPLVI	BIP208_2/OSIP208_2	4L0Q	A	0.004319	0.059352533	-49.29710	5.96190	0.5962
MKKEDIGRRF	BIP38_2/OSIP43_2	3CQR	B	0.039490	0.164618986	-17.09600	5.79540	0.5795
MLLSHLFASL	BIP50_3	2J3I	B	0.066400	0.220555451	-26.87000	5.49520	0.5495
NFHLPQP	BIP171_2/OSIP158_2	2Q4E	B	0.000497	0.043062533	1.04790	3.12320	0.3123
NLCQAYIVLH	BIP124_5/OSIP115_5	5FDN	A	0.031000	0.143811587	-15.29500	3.20230	0.3202
NTLKHNITLE	BIP222_1/OSIP222_1	5KOR	A	0.006225	0.067472018	-33.55820	5.48700	0.5487
NTYLPIVSYR	BIP244_9/OSIP244_9	1XJ5	C	0.005177	0.063126435	-15.36600	6.87130	0.6871
PTIVPDCLSK	BIP235_4/OSIP235_4	5GIJ	B	0.001978	0.048277539	-14.58880	5.35260	0.5353
PVYRQMAQFT	BIP90_3	1T1H	A	0.005057	0.062607232	-34.32460	7.07880	0.7079
QIKPQRT	BIP89_4	5GZZ	C	0.001022	0.043822401	-18.59600	5.54900	0.5549
QLCPSLNWGT	BIP31_1	2YIJ	A	0.041510	0.169276762	-26.04760	4.16530	0.4165
QLPFLITDSN	BIP22_1/OSIP25_1	3OGK	F	0.003555	0.055820995	-8.46930	7.72580	0.7726
QSFSVQLTTE	BIP62_3/OSIP58_3	4Z61	A	0.028800	0.138130201	11.37230	5.55480	0.5555
RCFPCHPS	BIP24_1	1RP0	A	0.004242	0.059034087	-30.52810	5.10340	0.5103
REESQQTKWV	BIP210_3/OSIP210_3	2VCE	A	0.057440	0.203277565	-17.08090	5.95410	0.5954
REWMVTPFVK	BIP239_5/OSIP239_5	2CDQ	B	0.008712	0.077115047	-3.47460	3.65290	0.3653
RGEEQIFWCQ	BIP227_1/OSIP227_1	4O7G	A	0.005910	0.066195474	-22.46280	4.09960	0.4100

Ligand	SIP	PDB_match	PDB Chain	Raw *p*-values (PepSite2)	BH correction (FDR=0.25)	Average △G (kcal/mol)	Std. Dev.	Std. Err. of Mean
RHFLEQSRLP	BIP89_5	5JO2	B	0.036180	0.156730397	-13.78640	2.74220	0.2742
RIFNFPFRIK	BIP44_5/OSIP49_5	4QEO	A	0.005177	0.063126435	-34.32050	5.52370	0.5524
RIWSLQLKYT	BIP235_1/OSIP235_1	3H7R	A	0.025510	0.129363564	-68.46480	6.11110	0.6111
RKRRNNFSCS	BIP142_4/OSIP134_4	4N0G	B	0.056720	0.201827368	-3.93630	4.66860	0.4669
RLQHHAESLP	BIP208_6/OSIP208_6	1N7G	B	0.034070	0.151535625	-10.33770	3.51080	0.3511
RPGLRPNGPS	OSIP77_2	1WE9	A	0.001697	0.046881324	-19.93050	5.21160	0.5212
RQFKALYQYI	BIP4_2/OSIP5_2	5HH7	A	0.022670	0.121560274	-54.42800	6.81270	0.6813
RRKSTNQMGL	BIP210_2/OSIP210_2	4TNM	A	0.001552	0.046163729	-29.94440	8.55490	0.8555
RRYRFPNSCL	BIP226_4/OSIP226_4	5HTR	A	0.028290	0.136803225	-31.41360	4.94000	0.4940
RYCMRLMMLN	BIP86_6	3PYA	A	0.000612	0.043062533	-40.24270	7.83410	0.7834
SDLQKDSWVQ	BIP90_3	4E2Q	C	0.003335	0.054807220	-7.11210	3.20690	0.3207
SFPNSNSGKV	BIP31_5	2FGE	A	0.003409	0.055150462	-1.52640	5.57090	0.5571
SGLSNTALAR	BIP229_2/OSIP229_2	2Q3O	B	0.004753	0.061280118	-35.22620	4.54440	0.4544
SILFFPLCKF	BIP171_6/OSIP158_6	5KSD	B	0.000252	0.043062533	-23.31720	4.25180	0.4252
SISKACERIP	OSIP4_4	2Q4X	B	0.000122	0.043062533	-56.71780	9.91560	0.9916
SLISVGLTKN	BIP224_3/OSIP224_3	2G5W	B	0.009518	0.080067059	-48.92090	5.36950	0.5370
SLLPFSLTDF	BIP237_2/OSIP237_2	5IGO	A	0.032290	0.147101005	-25.57600	4.95240	0.4952
SLQP	BIP213_1/OSIP213_1	5LAL	B	0.000271	0.043062533	-47.26680	6.35810	0.6358
SMFFFFLY	BIP105_2	3EI6	B	0.007954	0.074310897	-32.28160	5.69890	0.5699
SNLLYLATVT	BIP239_2/OSIP239_2	1MVL	A	0.045360	0.177952663	-16.96080	4.23500	0.4235
SNRLTMTGSK	BIP154_1/OSIP145_1	4NC4	C	0.011760	0.087958000	-36.54840	4.53820	0.4538
SRWSLEIAVK	BIP175_4	2CF6	A	0.039230	0.164012724	-40.36650	6.85170	0.6852
SSDPVNIWKE	BIP235_4/OSIP235_4	5GQR	B	0.000358	0.043062533	-22.05720	4.18880	0.4189
SVITYPSCTQ	BIP209_2/OSIP209_2	2ZFD	A	0.033850	0.151003875	-43.33060	5.95220	0.5952
TFEQYWLPLL	OSIP51_2	4A0H	A	0.001652	0.046648252	-46.96320	5.50460	0.5505
TFQRNYECAS	BIP236_3/OSIP236_3	4N7Q	A	0.003182	0.054111335	-19.41610	4.24450	0.4244
TLVATPRCVS	BIP97_6	4QQR	B	0.034690	0.153090811	-40.88980	4.55950	0.4559
TQSVPFTCKS	BIP231_2/OSIP231_2	2P1N	B	0.001471	0.045756756	-31.13340	7.76680	0.7767
TRCITCHLRS	BIP106_6	4Q75	B	0.046460	0.180324403	-13.12610	7.66320	0.7663
TTDKLIPEHM	BIP154_1/OSIP145_1	4DNU	A	0.002210	0.049456892	-4.62690	5.00830	0.5008
VATRQSPRVP	BIP122_2	3VEM	C	0.035310	0.154590507	-14.16080	3.56960	0.3570

Ligand	SIP	PDB_match	PDB Chain	Raw *p* -values (PepSite2)	BH correction (FDR=0.25)	Average △G (kcal/mol)	Std. Dev.	Std. Err. of Mean
VFSYFMDNKR	BIP224_2/OSIP224_2	2VY2	A	0.027020	0.133442025	-25.28370	4.49340	0.4493
VGIVPRQKKV	OSIP70_3	1XQ1	A	0.002532	0.051075564	-10.24690	3.33670	0.3337
VKSPVPSRTK	BIP44_5/OSIP49_5	4XAE	A	0.018780	0.110280281	-39.76020	6.15190	0.6152
VLVNANLDVE	BIP228_1/OSIP228_1	2G0Q	A	0.008121	0.074908671	-14.50460	7.74300	0.7743
VMGPFSGPSE	OSIP168_1	4EQ4	A	0.049690	0.187261714	-22.56180	5.99490	0.5995
VNGFSLNLSK	OSIP114_6	1GCC	A	0.058450	0.205260426	-39.87140	4.59500	0.4595
VQEKVITSSL	OSIP50_2	5A5K	S	0.025430	0.129157373	-26.01160	4.40630	0.4406
VSLVTIPNNN	OSIP108_3	5HAD	A	0.001466	0.045740500	-11.95970	5.68870	0.5689
VVFGGKMLNC	BIP0_5	4TJV	A	0.072140	0.231072809	-2.25840	3.07840	0.3078
VWINQILEQH	BIP214_1/OSIP214_1	1YHY	A	0.002668	0.051725656	2.08000	3.72570	0.3726
YPVLDAVEGA	BIP47_6	3FY4	C	0.000136	0.043062533	-28.64810	5.36340	0.5363
