# Supplementary material for: Large-scale docking predicts that sORF-encoded peptides may function through protein-peptide interactions in Arabidopsis thaliana
Source: PLoS One. 2018 Oct 15;13(10):e0205179. doi: 10.1371/journal.pone.0205179 (PMC6188750; doi:10.1371/journal.pone.0205179)
Supplement: S3 Table — For fields that are indicated as monomers in Protein stoichiometry, the other chain in the structure is either a characterized peptide or the monomers may biologically aggregate to form dimers. (PDF) [file pone.0205179.s007.pdf]

Peptide with position	Sequence	PDB match	Other chain	Protein classification	Protein stoichiometry	△G (kcal/ mol)	raw *p*-values (PepSite2)	Peptide binding residues on protein-peptide model (PDB)
BIP44_5/ OSIP49_5;21-30	RIFNFPFRIK	4QEO(A)	P (substrate H3 peptide)	Transcription/ DNA	Monomer	-34.320500	0.005177	418, 420, 482, 485, 495, 496, 497, 498, 499, 534, 560, 562, 591, 592, 593, 594, 595, 596, 599, 600, 601, 602, 603
BIP47_6;15-24	YPVLDAVEGA	3FY4(C)	A	Lyase	Monomer	-28.648100	0.000136	162, 244, 245, 246, 247, 257, 292, 293, 301, 363, 364, 365, 368, 408, 415, 417, 420, 422
BIP237_2/ OSIP237_2;5-14	SLLPFSLTDF	5IGO(A)	U (Tribbles Homolog 1 peptide)	Hydrolase/ Peptide	Monomer	-25.576000	0.032290	373, 375, 390, 391, 422, 423, 441, 465, 467, 526, 528, 549, 550, 551, 554, 568, 592, 593, 594, 595, 596, 646
OSIP51_2;37-46	TFEQYWLPLL	4A0H(A)	B	Transferase	Homo 2-mer - A2	-46.963200	0.001652	326, 328, 331, 332, 369, 370, 473, 488, 489, 581, 587, 617, 618, 644, 761, 775, 777, 778
BIP103_3;28-37	FFDDVKSRRL	2XQR(A)	B	Hydrolase/ Inhibitor	Hetero 2-mer - AB	-29.553100	0.004392	20, 47, 48, 49, 50, 79, 80, 82, 101, 105, 107, 145, 146, 148, 203, 239, 242, 279
BIP208_6/ OSIP208_6;24-33	RLQHHAESLP	1N7G(B)	A	Lyase	Homo 4-mer - A4	-10.337700	0.034070	130, 134, 135, 168, 179, 180, 181, 187, 188, 190, 191, 193, 194, 195
BIP142_4/ OSIP134_4;14-23	RKRRNNFSCS	4N0G(B)	D	Hydrolase/ Receptor	Hetero 2-mer - AB	-3.936300	0.056720	262, 276, 277, 278, 279, 280, 281, 282, 286, 287, 288
BIP209_2/ OSIP209_2;12-21	SVITYPSCTQ	2ZFD(A)	B	Signaling protein/ Transferase	Hetero 2-mer - AB	-43.330600	0.033850	76, 77, 78, 85, 86, 89, 130, 131, 133, 139, 144, 147, 148, 151, 154, 171, 172, 175, 183, 191, 198, 201
BIP22_1/ OSIP25_1;19-28	QLPFLITDSN	3OGK(F)	S (JAZ1 incomplete degron peptide)	Protein binding	Hetero 16-mer - A8B8	-8.469300	0.003555	85, 89, 90, 91, 348, 350, 351, 386, 411, 412, 413, 414, 442, 444, 469, 472, 496, 521, 564
BIP229_2/ OSIP229_2;10-19	DLNQPKMYKF	2XCM(F)	B	Chaperone/ Protein binding	Hetero 3-mer - ABC	-19.530600	0.024810	158, 159, 160, 161, 162, 163, 164, 165, 166, 179, 180, 181, 185, 187, 194, 204, 207, 208, 210, 211, 212, 213, 221
BIP231_6/ OSIP231_6;18-27	ILRRICILIT	4A0G(A)	B	Transferase	Homo 2-mer - A2	-50.443500	0.002503	324, 325, 326, 327, 328, 369, 370, 473, 476, 481, 488, 489, 494, 587, 775, 776, 777, 778, 782
BIP244_1/ OSIP244_1;33-42	IFLFFWFHAL	4N0G(D)	B	Hydrolase/ Receptor	Hetero 2-mer - AB	-39.596300	0.029950	70, 71, 72, 73, 74, 98, 99, 100, 101, 132, 135, 136, 139, 140, 142, 143, 144, 146, 147, 149, 150
BIP244_9/ OSIP244_9;2-11	NTYLPIVSYR	1XJ5(C)	B	Transferase	Homo 4-mer - A4	-15.366000	0.005177	239, 264, 265, 266, 267, 268, 272, 273, 274, 317, 318, 320, 321, 322, 323, 324, 325
OSIP50_2;34-43	VQEKVITSSL	5A5K(S)	I	Transferase	Homo 6-mer - A6	-26.011600	0.025430	9, 11, 12, 13, 14, 15, 36, 37, 38, 41, 55, 112, 116, 119, 120, 124, 125, 128, 130, 179
BIP243_2/ OSIP243_2;19-28	LNHHCRFSGK	5E4W(D)	A	Transport protein	Hetero 2-mer - AB	-10.308400	0.035250	267, 268, 269, 270, 271, 272, 291, 292, 293, 294, 295, 296, 297, 298
