# Supplementary material for: Large-scale docking predicts that sORF-encoded peptides may function through protein-peptide interactions in Arabidopsis thaliana
Source: PLoS One. 2018 Oct 15;13(10):e0205179. doi: 10.1371/journal.pone.0205179 (PMC6188750; doi:10.1371/journal.pone.0205179)
Supplement: S4 Table — Empty fields in the ligand column indicate only catalytic sites and no known ligand is known to bind at the respective sites. (PDF) [file pone.0205179.s008.pdf]

Peptide with position	Sequence	PDB match	PDB Chain	Classification	ΔG (kcal/mol)	raw *p*-values (PepSite2)	Peptide binding residues on protein-peptide model (PDB)	Ligand binding sites (PDBeMotif) / Catalytic site (PDB)	Ligand (Chain)
BIP3_1;3-12	LEKVVENTVA	4BQE	A	Transferase	-27.0502	0.01141	91, 136, 285, 297, 343, 382, 384, 385, 573, 575, 576, 577, 617, 620, 621, 622, 623, 773, 774, 778, 779	575, 617, 620	TRIETHYLENE GLYCOL (A)
BIP227_1/OSIP227_1;20-29	RGEEQIFWCQ	4O7G	A	Oxidoreductase	-22.4628	0.00591	89, 92, 96, 99, 103, 113, 114, 115, 116, 119, 120, 121, 123, 124, 126, 127, 130, 133, 134, 137, 138, 142, 143	29, 48, 51, 52, 55, 56, 59, 103, 106, 113, 114, 116, 118, 119, 122, 171, 172, 175, 176, 178, 179	HEME (A)
BIP231_3/OSIP231_3;25-34	LLTSYELSCV	4PYH	A	Hydrolase	-15.7929	0.004998	87, 88, 133, 135, 136, 139, 165, 166, 167, 277, 278, 279, 280, 281, 314, 328, 330	307, 314, 330, 167, 278, 314, 332	ALPHA-D-GLUCOSE (A)
BIP89_4;33-42	QIKPQRT	5GZZ	C	Ligase/ Transferase	-18.596	0.001022	8, 9, 32, 33, 34, 35, 36, 37, 38, 39, 40, 41, 42, 43, 45, 55, 111, 204, 205, 206, 207	8, 13, 54, 55, 67	GLUTATHIONE (C)
BIP239_2/OSIP239_2;21-30	SNLLYLATVT	1MVL	A	Lyase	-16.9608	0.04536	74, 77, 78, 81, 82, 83, 84, 88, 90, 118, 119, 120, 121, 122, 123, 124	90, 175	-
BIP224_3/OSIP224_3;12-21	SLISVGLTKN	2G5W	B	Oxidoreductase	-48.9209	0.009518	74, 108, 138, 139, 140, 141, 147, 150, 191, 243, 245, 246, 282, 283, 284, 321, 322, 344, 367, 371	186, 189, 191, 245, 371 / 189, 191, 239, 271	DIEN-1-OIC ACID (B)
OSIP108_3;12-21	VSLVTIPNNN	5HAD	A	Photosynthesis	-11.9597	0.001466	690, 694, 711, 714, 715, 717, 718, 721, 729, 731, 758, 774, 776, 777, 778, 779, 796, 798, 799, 800	694, 715, 718, 729, 731	ETHYLENE GLYCOL (A)
BIP0_3;28-37	KDLTFFPPKN	3RIZ/4LSA	A	Signaling protein	-17.4497	0.002727	563, 564, 597, 599, 615, 641, 642, 643, 644, 645, 646, 647, 648, 649, 657, 681, 705, 729	563, 564, 597, 599, 601, 642, 646, 647, 657, 681, 705, 729	BRASSINOLIDE (A)
BIP175_4;20-29	SRWSLEIAVK	2CF6	A	Oxidoreductase	-40.3665	0.03923	53, 58, 60, 113, 114, 115, 116, 117, 119, 121, 122, 123, 273, 274, 275, 276, 277, 295, 296, 297, 298, 299	48, 49, 167, 188, 189, 190, 191, 192, 211, 212, 216, 251, 252, 253, 274, 275, 298, 299, 300	NADP (A)
BIP213_1/OSIP213_1;30-39	SLQP	5LAL	B	Plant protein	-47.2668	0.0002713	51, 73, 74, 75, 76, 78, 104, 106, 110, 111, 112, 113, 139, 140, 144, 166, 168, 169, 170, 171, 172, 173, 174, 175	108, 112, 113, 138, 139, 140, 106, 114, 116, 137, 139, 169, 175	GLYCINE (B)
BIP244_8/OSIP244_8;17-26	HQNQIKEYFI	4HHD	B	Transferase	-11.4099	0.07966	474, 475, 476, 499, 541, 555, 574, 575, 576, 577, 578, 579, 589, 590, 593, 594, 595, 596, 597, 598, 600, 601, 604	478, 480, 487, 511, 512, 513, 515, 516, 525, 528, 529, 532, 542, 544, 556, 558, 571, 573, 575	FLAVIN MONONUCLEOTIDE (B)
BIP206_2/OSIP206_2;26-35	EDVSTRYNIL	1U1J	A	Transferase	-49.2971	0.05147	437, 439, 490, 567, 607, 647, 649, 650, 671, 672, 674, 699, 700, 701, 732, 733, 734, 736, 737	18, 116, 118, 517, 521, 522, 523, 567	5-METHYL-5,6,7,8-TETRAHYDROFOLIC ACID (A)
BIP44_5/OSIP49_5;21-30	RIFNFPFRIK	4QEO	A	Transcription/ DNA	-34.3205	0.005177	418, 420, 482, 485, 495, 496, 497, 498, 499, 534, 560, 562, 591, 592, 593, 594, 595, 596, 599, 600, 601, 602, 603	456, 457, 458, 492, 493, 548, 551, 552, 593, 611, 612, 613, 614	S-ADENOSYL-L-HOMOCYSTEINE (A)
BIP235_4/OSIP235_4;13-22	EPTIVPDCLS	2Q4W	A	Oxidoreductase	-13.905	0.03904	41, 45, 46, 49, 50, 52, 65, 67, 92, 93, 94, 111, 113, 139, 141	54, 91, 93, 94, 95, 96, 97, 101, 102, 112, 161, 162, 166, 167, 169, 170, 171, 173, 177, 227, 228, 382, 479, 480, 514, 517 / 275, 162, 96	FLAVIN-ADENINE DINUCLEOTIDE (A)
BIP209_1/OSIP209_1;31-40	KHSKCKYAMQ	4EET	B	Signaling protein flavoprotein	-14.2823	0.04938	386, 387, 388, 389, 390, 391, 393, 405, 407, 409, 475, 486, 488	392, 394, 401, 425, 426, 427, 429, 430, 439, 442, 443, 446, 456, 458, 468, 470, 472, 485, 486, 487, 489	FLAVIN MONONUCLEOTIDE (B)
BIP105_2;29-38	SMFFFFLY	3EI6	B	Transferase	-32.2816	0.007954	37, 39, 40, 43, 46, 60, 63, 64, 65, 129, 152, 155, 209, 237, 239, 240, 267, 269, 270, 278, 328, 368	37, 39, 63, 64, 127, 128, 129, 152, 205, 209, 237, 240, 267, 269, 270, 278, 364, 404	(2S,6S)-2-AMINO-6-[({3-HYDROXY-2-METHYL-5-[(PHOSPHONOOXY)METHYL]PYRIDIN-4-YL}METHYL)AMINO]HEPTANEDIOIC ACID (B)
OSIP68_6;38-47	GIYLYNKCAF	3R0Q	G	Transferase	-31.8432	0.003906	37, 38, 39, 41, 43, 44, 47, 48, 143, 145, 147, 151, 152, 239, 321, 322, 323, 325, 356, 383	36, 39, 45, 48, 54, 78, 79, 99, 100, 101, 126, 127, 128, 129, 143, 154, 157	S-ADENOSYL-L-HOMOCYSTEINE(G)
OSIP51_2;37-46	TFEQYWLPLL	4A0H	A	Transferase	-46.9632	0.001652	326, 328, 331, 332, 369, 370, 473, 488, 489, 581, 587, 617, 618, 644, 761, 775, 777, 778	326, 369, 370, 473, 587, 775, 777	7-KETO-8-AMINOPELARGONIC ACID (A)
BIP229_2/OSIP229_2;32-41	SGLSNTALAR	2Q3O	B	Oxidoreductase	-35.2262	0.004753	30, 31, 32, 33, 64, 74, 108, 138, 139, 141, 150, 186, 189, 191, 245, 343, 344, 371	33, 35, 186, 189, 191, 239, 244, 271	-

Peptide with position	Sequence	PDB match	PDB Chain	Classification	$\triangle G$ (kcal/mol)	raw *p*-values (PepSite2)	Peptide binding residues on protein-peptide model (PDB)	Ligand binding sites (PDBeMotif) / Catalytic site (PDB)	Ligand (Chain)
BIP235_1/OSIP235_1;15-24	RIWSLQLKYT	3H7R	A	Oxidoreductase	-68.4648	0.02551	24, 25, 47, 48, 79, 111, 121, 122, 178, 202, 203, 207, 208, 209, 210, 286, 287, 288, 289, 291, 292	22, 23, 24, 43, 48, 77, 110, 154, 155, 176, 202, 203, 204, 205, 206, 207, 208, 214, 235, 250, 251, 252, 53, 254, 258, 261, 262 / 77, 43, 48, 110	NADP (A)
BIP86_6;6-15	RYCMRLMMLN	3PYA	A	Isomerase	-40.2427	0.000612	205, 206, 208, 209, 241, 245, 248, 260, 263, 264, 287, 369, 377, 379, 380, 412, 415, 417, 418, 463, 464, 508	208, 209, 245, 260, 263, 329, 333, 369, 412, 417, 463, 464, 505	S-[(2E,6E,10E)-14-(DIMETHYLAMINO)-3,7,11-TRIMETHYLTETRADECA-2,6,10-TRIEN-1-YL] TRIHYDROGEN THIODIPHOSPHATE (A)
BIP103_3;28-37	FFDDVKSRRL	2XQR	A	Hydrolase/ Inhibitor	-29.5531	0.004392	20, 47, 48, 49, 50, 79, 80, 82, 101, 105, 107, 145, 146, 148, 203, 239, 242, 279	22, 23, 39, 47, 82, 83, 148, 149, 203, 204, 279	FRUCTOSE(A)
BIP142_3_OSIP134_3;10-19	LAEDTFGEIS	2VTB	D	Lyase/DNA	-48.017	0.001674	193, 194, 195, 198, 199, 273, 317, 320, 321, 324, 328, 434, 441, 443, 444, 446	325, 328, 391	-
BIP222_1/OSIP222_1;37-46	NTLKHNITLE	5KOR	A	Transferase	-33.5582	0.006225	180, 181, 182, 183, 184, 271, 300, 301, 366, 368, 481, 483, 501, 523, 524, 551, 552, 553	181, 183, 184, 366, 416, 417, 418, 459, 463, 466, 482, 483, 484	GUANOSINE-5'-DIPHOSPHATE (A)
BIP214_1/OSIP214_1;63-72	VWINQILEQH	1YHY	A	Transferase	2.08	0.002668	177, 181, 184, 377, 485, 486, 489, 507, 508, 509, 510, 511, 512, 513, 568, 570, 571, 572, 574, 575, 582, 583, 584, 585, 586	485, 486, 487, 488, 513, 537, 538, 539, 540, 565, 567, 568, 569, 570 / 121, 144, 123, 167, 485, 513, 570, 571, 207, 206, 199, 198, 511	ETHYL DIHYDROGEN DIPHOSPHATE & METSULFURON METHYL & FAD (A)
BIP47_6;15-24	YPVLDAVEGA	3FY4	C	Lyase	-28.6481	0.0001355	162, 244, 245, 246, 247, 257, 292, 293, 301, 363, 364, 365, 368, 408, 415, 417, 420, 422	298, 301, 364	-
BIP44_5/OSIP49_5;39-48	VKSPVPSRTK	4XAE	A	Oxidoreductase	-39.7602	0.01878	138, 140, 147, 153, 214, 216, 232, 233, 234, 235, 236, 237, 238, 278, 282, 309, 333, 339, 341, 342, 343	235, 237, 293	SODIUM ION (A)
BIP34_4/OSIP39_4;2-11	KTGDAYLSSG	1XM8	B	Hydrolase	-46.635	0.02615	12, 54, 55, 56, 57, 58, 59, 60, 61, 64, 83, 84, 85, 112, 113, 131, 134, 138, 139, 140, 142, 143, 169, 171, 248	134, 137, 138, 140, 171, 248, 251, 54, 56, 112 / 58	ACETIC ACID & Zn (B)
BIP212_2/OSIP212_2;31-40	FAVEFQVRK	5D79	B	Oxidoreductase	-49.8954	0.004563	113, 174, 175, 176, 291, 299, 301, 344, 345, 346, 359, 361, 362, 363, 395, 397, 399, 428, 530, 432	106, 107, 108, 109, 110, 111, 112, 113, 117, 130, 149, 174, 177, 178, 180, 181, 182, 187, 188, 233, 234, 237, 238, 239, 470, 472, 473, 474	FLAVIN-ADENINE DINUCLEOTIDE (B)
OSIP94_4;15-24	DEYDCWWSS	2HJ3	A	Oxidoreductase	-37.8687	0.01852	11, 12, 15, 16, 17, 19, 20, 23, 86, 89, 90, 91, 93, 95, 96, 98, 99, 102, 105, 106, 107	11, 15, 16, 18, 19, 23, 52, 56, 57, 60, 61, 83, 86, 87, 89, 90, 93, 95, 98, 103, 107	FLAVIN-ADENINE DINUCLEOTIDE (A)
